# Copper Complexes with N,N,N-Tridentate Quinolinyl Anilido-Imine Ligands: Synthesis and Their Catalytic Application in Chan−Lam Reactions

**DOI:** 10.3390/molecules28217406

**Published:** 2023-11-03

**Authors:** Xiaoyu Zhou, Jiaxin Yang, Zhiqiang Hao, Zhangang Han, Jin Lin, Guo-Liang Lu

**Affiliations:** 1 National Experimental Chemistry Teaching Center, Hebei Key Laboratory of Organic Functional Molecules, College of Chemistry and Materials Science, Hebei Normal University, Shijiazhuang 050024, China; 2 Auckland Cancer Society Research Centre, Faculty of Medical and Health Sciences, Maurice Wilkins Centre, The University of Auckland, Auckland 1142, New Zealand

**Keywords:** anilido-imine ligands, copper complexes, benzimidazole, Chan–Lam coupling

## Abstract

The treatment of 2-(ArNC(H))C_6_H_4_-HNC_9_H_6_N with *n*-BuLi and the subsequent addition of CuCl_2_ afforded the anilido-aldimine Cu(II) complexes **1**-**5** Cu[{2-[ArN=C(H)]C_6_H_4_}N(8-C_9_H_6_N)]Cl (Ar = 2,6-*^i^*Pr_2_C_6_H_3_ (**1**), 2,4,6-(CH_3_)_3_C_6_H_2_ (**2**), 4-OCH_3_C_6_H_4_ (**3**), 4-BrC_6_H_4_ (**4**), 4-ClC_6_H_4_ (**5**)), respectively. All the copper complexes were fully characterized by IR, EPR and HR-MS spectra. The X-ray diffraction analysis reveals that **2** and **4** are mononuclear complexes, and the Cu atom is sitting in a slightly square-planar geometry. These Cu(II) complexes have exhibited excellent catalytic activity in the Chan–Lam coupling reactions of benzimidazole derivatives with arylboronic acids, achieving the highest yields of up to 96%.

## 1. Introduction

**Scheme 1:**
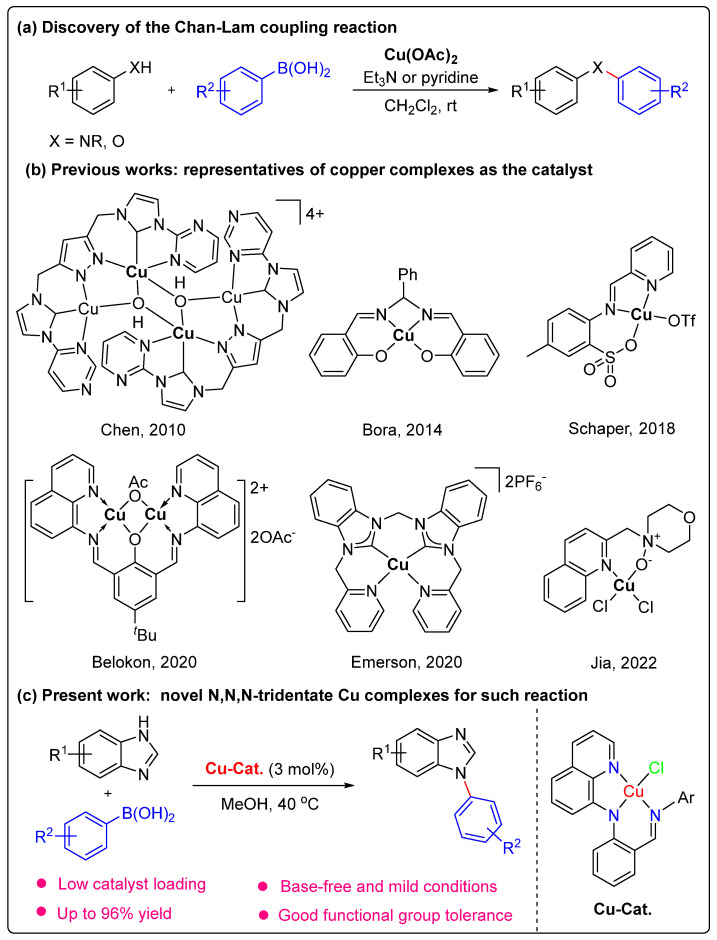
Classical and selected Cu complexes for Chan–Lam reactions [15,16,17,19,20,21].

The construction of C-N bonds is one of the most widely practiced reactions in synthetic chemistry. The name reactions, such as the Buchwald–Hartwig [1,2], Chan–Lam [3,4] and Ullmann [5] reactions, have been extensively explored for C-N bond formation. These reactions have a wide range of applications, such as in pesticide synthesis and pharmaceutical and material chemistry [6]. Among these reactions, the Cu-promoted Chan–Lam coupling reaction features the advantages of low cost and operational simplicity and is an efficient synthetic method to construct various carbon-heteroatom bonds. In 1998, this name reaction was first reported to construct C-X bonds through the coupling between arylboronic acids and different nucleophiles in the presence of Cu(OAc)_2_ as a catalyst (Scheme 1a) [7,8,9]. Since then, Cu and other transition metal-catalyzed Chan–Lam reactions have contributed greatly to C-N bond formation [10,11,12,13].

In addition to metal salts, well-defined copper complexes were also rapidly developed as catalysts for these reactions. Representative examples can be found in Scheme 1b [14,15,16,17,18,19,20,21]. In 2000, Collman and co-workers used 10 mol% of simple Cu complex [Cu(OH)·TMEDA]_2_Cl_2_ (TMEDA = N,N,N′,N′-Tetramethylethylenediamine) to produce a variety of *N*-arylimidazoles with good-to-excellent yields by cross-coupling arylboronic acids with imidazole compounds [14]. In 2018, Schaper et al. synthesized a sulfonato-imino copper(II) complex that can efficiently catalyze the *N*-arylation or *N*-alkylation of amines and anilines with arylboronic acids without the need for any bases [18]. Recently, Emerson’s group reported a new tetradentate NHC-copper(II) complex, which showed good catalytic activity in the Chan–Lam coupling of aniline and phenylboronic acid [20]. More recently, Jia et al. developed an efficient method for the copper-promoted Chan–Lam coupling of 1*H*-imidazole derivatives with arylboronic acids using an N, O-bidentate Cu complex (8.0 mol%) as a catalyst [21]. Although considerable progress has been made in this field, there are still limitations, such as long reaction times, high catalyst loading, or the use of a base. Thus, developing novel and efficient catalysts for Chan–Lam coupling under mild conditions is in high demand.

Moreover, it is well-accepted that ligands play an important role in catalysis. They can improve the solubility of transition-metal complexes in organic media and modify catalytic activity via the precise control of their electronic and geometric properties [22]. Recently, our group has been focusing on synthesizing anilido-imine ligand-supported transition-metal complexes. The corresponding Ni [23], Fe [24], Cr [25] and Cu [26] complexes exhibit moderate to high activities in olefin polymerization and the atom transfer radical polymerization (ATRP) reaction, but studies on organic transformation are less reported. Herein, we report the synthesis of several copper complexes bearing N,N,N-tridentate anilido-imine ligands, which displayed excellent catalytic performance in the Chan–Lam coupling of arylboronic acids with benzimidazole derivatives under base-free conditions (Scheme 1c).

## 2. Results and Discussion

### 2.1. Synthesis and Characterization of Ligands and Cu(II) Complexes

**Scheme 2:**
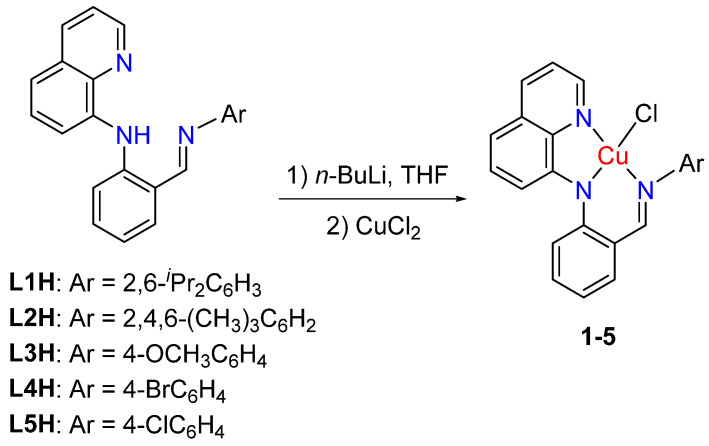
Synthesis of complexes **1**-**5**.

The tridentate anilido-imine ligands **L1H**, **L2H**, **L4H**, **L5H** and Cu(II) complex **1** were prepared according to the literature method [27] and our recent reports [26,28]. The ligand **L3H** and other Cu complexes **2**-**5** were synthesized following the analogous methods. Cu(II) complex **1** was prepared using the literature method [26]. The other Cu complexes (**2**-**5**) were synthesized following the analogous methods. The reactions of ligands **L2H**-**L5H** with *n*-BuLi at −78 °C followed by adding CuCl_2_ afforded complexes **2**-**5** as brown powders in 55–68% yields, respectively (Scheme 2). These complexes are air- and moisture-stable and soluble in common organic solvents (e.g., THF, CH_2_Cl_2_ and toluene). Complexes **2**-**5** were all characterized by HR-MS, EPR (electron paramagnetic resonance) and IR spectroscopy. The HR-MS peaks at 427.1104 (**2**), 415.0740 (**3**), 462.9740 (**4**) and 419.0245 (**5**) match well with the calculated masses of the cationic species [M-Cl]^+^ (Figures S1–S4). The EPR spectra of complexes **2**-**5** exhibit the values of g_⊥_ around 2.04–2.09 and g_║_ around 2.11–2.16 (Figure S5), which are comparable to the EPR data of some reported mononuclear Cu complexes [29] and our previous work [30]. In their IR spectra, a strong vibration band at 1602–1613 cm^−1^ can be assigned to the imine group. The signals between 3100 and 3500 cm^−1^ of the N-H group in the ligands disappeared in the IR spectra of complexes **2**-**5**, indicating the Cu-N bond formation through the deprotonation of the –NH group.

### 2.2. Description of the Crystal Structures of Complexes ***2*** and ***4***

**Figure 1:**
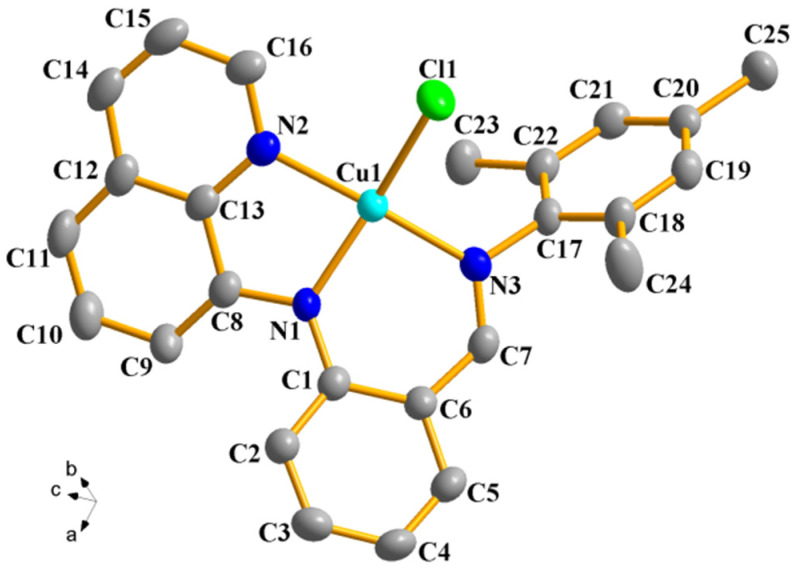
Molecular structure of Cu complex **2** (CCDC: 2248465) with thermal ellipsoids drawn at a 50% probability level. Hydrogen atoms are omitted for clarity. Selected bond lengths (Å): Cu(1)-N(1) 1.927(3), Cu(1)-N(2) 1.990(3), Cu(1)-N(3) 1.973(3), Cu(1)-Cl(1) 2.2248(12), N(1)-C(8) 1.389(5), N(2)-C(13) 1.367(5), N(3)-C(7) 1.288(5), N(3)-C(17) 1.442(5); Selected bond angles (◦):C(1)-N(1)-C(8) 122.1(3), N(1)-Cu(1)-Cl(1) 159.97(11), N(1)-C(1)-C(6) 119.2(4), N(3)-Cu(1)-N(2) 158.12(11), C(8)-N(1)-Cu(1) 114.2(3).

**Figure 2:**
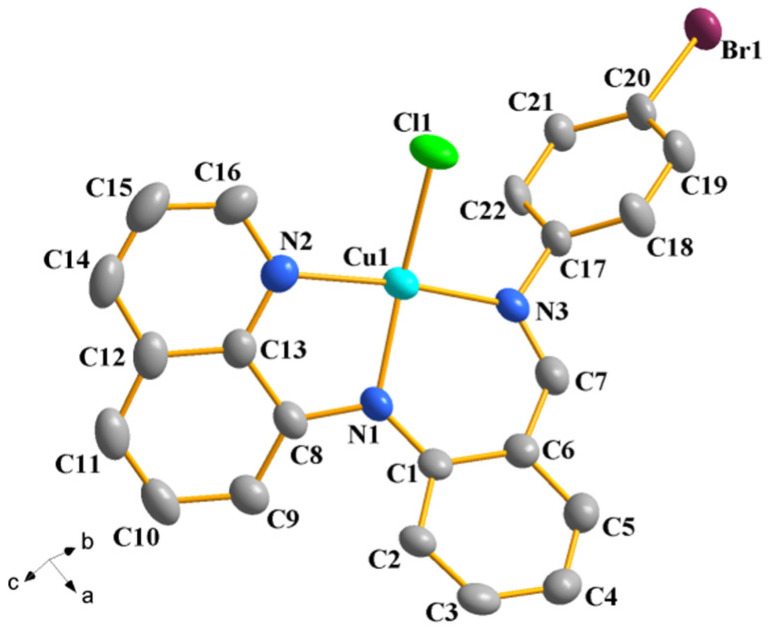
Molecular structure of Cu complex **4** (CCDC: 2248467) with thermal ellipsoids drawn at a 50% probability level. Hydrogen atoms are omitted for clarity. Selected bond lengths (Å) Cu(1)-N(1) 1.956(4), Cu(1)-N(2) 1.985(4), Cu(1)-N(3) 1.980(4), Cu(1)-Cl(1) 2.2529(13), N(1)-C(8) 1.393(5); N(2)-C(13) 1.355(6); N(3)-C(7) 1.295(6), N(3)-C(17) 1.427(6); Selected bond angles (◦):C(1)-N(1)-C(8) 122.6(4), N(1)-Cu(1)-Cl(1) 161.71(12), N(1)-Cu(1)-N(3) 90.90(15), N(1)-C(1)-C(6) 120.0(4), N(3)-Cu(1)-Cl(1) 94.30(11).

Crystals **2** and **4** suitable for single-crystal X-ray diffraction were grown from their solutions in CH_2_Cl_2_/hexane. X-ray single-crystal diffraction analyses show that crystals **2** and **4** are monoclinic with space groups of *C*c and *P*2_1_/*c*, respectively. The crystal data are summarized in Table 1. Their molecular structures are depicted in Figure 1 and Figure 2, with selected bond lengths and angles. The unit cell of **4** contains two crystallographically-independent molecules with similar connectivity and only one molecule is depicted in Figure 2. The central metal Cu is coordinated by three nitrogen atoms and one chlorine atom, and the environment around the metal can be described as a distorted square-planar. The Cu-Cl bond lengths (2.2248(12) Å for **2** and 2.2529(13) Å for **4**) are relatively longer than those of 2.1997(13)–2.2001(13) Å in complex [CuCl_2_(Mes-BIAN)] OEt_2_ [31]. The Cu-N_amino_ bond lengths of **2** and **4** are 1.927(3) Å and 1.956(4) Å, respectively, which are similar to the Cu-N_amino_ bond length (1.939(3) Å) in copper complex **1 [26]**. The Cu-N_imino_ bond lengths (1.973(3) Å for **2** and 1.980(4) Å for **4**) are slightly longer than that of **1** (1.967(3) Å) but much shorter than the bond length of 2.026(1) Å in the sulfonato-imine copper complex [18].

### 2.3. Catalytic Activity

In order to investigate the catalytic properties of copper complexes **1**-**5** in the Chan–Lam coupling reaction, benzimidazole **6a** and phenylboronic acid **7a** were set as the model substrates. The reaction parameters, such as solvents, reaction time, temperature and catalyst loading, were optimized using complex **1** as a catalyst, and the catalytic results are shown in Table 2. At first, the solvent effect was investigated using 5 mol% of complex **1** under an air atmosphere and base-free conditions. The results showed that the best catalytic activity was achieved when methanol was used as the solvent to give the target product **8a** with 90% yield at 50 °C for 20 h (Table 2, entry 1). Using other solvents, such as *^i^*PrOH, 1,4-Dioxane, DMF, CH_3_CN, THF, and toluene, under the same conditions gave only trace or low yields (Table 2, entries 2–7). Next, the reaction time and temperature were screened. When the reaction time was shortened from 20 h to 16 h and 12 h, the yields of **8a** were 92% and 90%, respectively (Table 2, entries 8 and 9). Further reducing the reaction time to 8 h slightly decreased the yield to 83% (Table 2, entry 10). Since the yields were almost the same at 20 h and 12 h, 12 h was considered the optimized reaction time. Upon reducing the reaction temperature from 50 °C to 40 °C, the yield of **8a** increased to 92% (Table 2, entry 11). When the reaction was performed either at 60 °C or room temperature, the yields of desired products dropped slightly (Table 2, entries 12 and 13). Thus, the best reaction temperature was selected to be 40 °C. Afterward, the effect of catalyst loading on the reaction was explored. Changing the catalyst loading of **1** from 5 mol% to 3 mol%, 2 mol%, and 1 mol% resulted in yields of 93%, 84%, and 71%, respectively (Table 2, entries 14–16). Therefore, the most suitable catalyst loading was decided to be 3 mol%. Subsequently, the catalytic activities of catalysts **2**-**5** were tested under the optimized conditions, and complex **3** showed the highest activity, furnishing product **8a** with 96% yield (Table 2, entries 17–20). The CuCl_2_ was also tested in the Chan–Lam coupling of **6a** and **7a** to afford the corresponding product with only 48% yield, showing the important role of the ligand in the catalytic reaction (Table 2, entry 21). The control experiment indicated that the coupling reaction did not occur in the absence of any catalyst (Table 2, entry 22). Finally, when the coupling reaction was performed under an inert atmosphere, no product **8a** was observed, indicating the key role of air in the catalytic reaction (Table 2, entry 23).

We next examined the substrate generality of this coupling reaction (Table 3). The phenylboronic acids with electron-deficient and electron-rich groups efficiently proceeded to afford the desired products (**8a**–**8p**) in 75–96% yields. Various substituents, including methyl, methoxyl, fluoro, chloro, bromo, phenyl, aceto, ester and cyano, were tolerated, indicating good functional group compatibility. The steric effect has relatively little influence on this coupling reaction, which can be seen from **8b**–**8d** and **8g**–**8h**. However, heteroaryl boronic acids such as furan-2-ylboronic acid and thiophen-2-ylboronic acid were not suitable for the present catalytic system, and only trace amounts of the desired products—**8q** and **8r**—were obtained. Gratifyingly, disubstituted 1,4-phenylenediboronic acid was also smoothly coupled with benzimidazole **6a** to produce the corresponding **8s** in 76% yield. Further, the reaction of **6** with different monosubstituted and disubstituted groups with phenylboronic acid gave the corresponding products in good-to-excellent yields (**8t**–8**y**). The reaction between dimethylbenzimidazole and F- or OMe-substituted phenylboronic acid also afforded the corresponding products, **8x** and **8y**, in 88% and 90% yields, respectively.

**Scheme 3:**
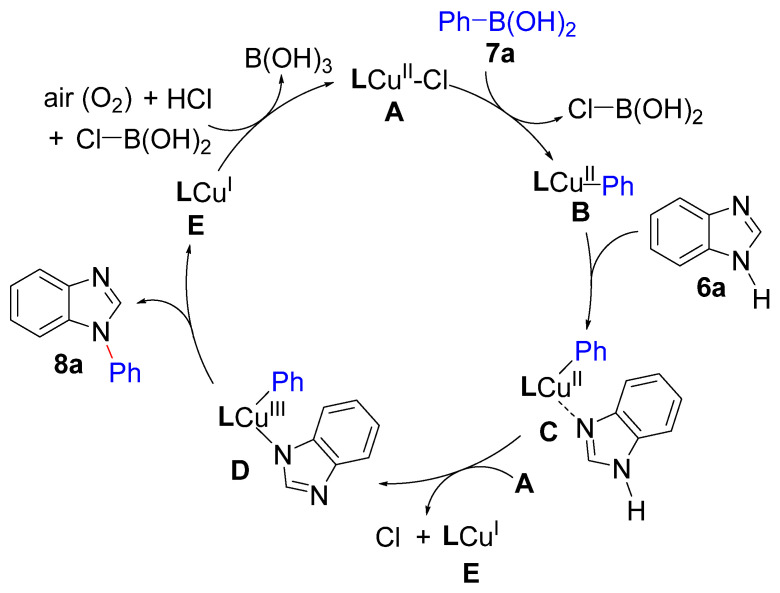
Proposed catalytic mechanism.

Taking previous reports [19,32,33,34] into account, a plausible mechanism for this Cu-catalyzed Chan–Lam reaction is depicted in Scheme 3. Initially, phenylboronic acid **7a** undergoes a transmetallation reaction with Cu^II^ complex **A** to produce Cu^II^ species **B** and ClB(OH)_2_. Species **B** reacts with benzimidazole **6a** to form a Cu^II^ intermediate **C**, which lowers the Cu^III^/Cu^II^ reduction potential [35]. Then, intermediate **C** undergoes a disproportionation process [18,36,37,38] in the presence of **A** to afford the high-valence Cu^III^ intermediate **D** and Cu^I^ species **E**, and a molecule of HCl is released. Subsequently, intermediate **D** yields the product 1-phenylbenzimidazole **8a** and generates another molecule of intermediate **E** through a reductive elimination step. Finally, intermediate **E** is converted to Cu^II^ complex **A** in the presence of air, HCl and ClB(OH)_2_ to finish the catalytic cycle.

## 3. Materials and Methods

### 3.1. General Considerations

All manipulations (except the catalytic reactions) under a nitrogen atmosphere were carried out using a Schlenk line. THF was distilled from Na and benzophenone under N_2_ before use. The other solvents for the catalytic reactions, CuCl_2_ and other reagents were obtained from commercial suppliers and used without further purification. IR spectra were recorded as KBr disks on a Thermo Fisher iS50 spectrometer (Thermo Fisher, Waltham, MA, USA). Mass spectroscopy was performed with an AB SCIEX 3200 Q-TRAP mass spectrometer (AB SCIEX, Framingham, MA, USA). The melting points were determined on an X-5 melting point apparatus (Beijing Tech Instrument Co., Ltd., Beijing, China). High-resolution mass spectra (HR-MS) were acquired using an Agilent 6210 ESI-TOF mass spectrometer (Agilent technology Co., Ltd, Santa Clara, CA, USA). ^1^H NMR and ^13^C{^1^H} NMR spectra were recorded on Zhongke-Niujin Quantum-I 400 MHz spectrometer (Zhongke-Niujin Co., Ltd., Wuhan, China). The EPR spectra were obtained at 77K in CH_2_Cl_2_ (0.01 M) solution using a Bruker-A200 Electron Spin Paramagnetic Resonance instrument (Bruker Corporation, Karlsruhe, Germany).

### 3.2. X-ray Crystallographic Studies

Diffraction data of **2** and **4** were collected on an Oxford Diffraction SuperNova dual source diffractometer with graphite-monochromated Cu-*K*α radiation (λ = 1.54178 Å). The structures were solved by direct methods and refined by full-matrix least-squares on *F*^2^. All non-hydrogen atoms were refined anisotropically. The hydrogen atoms were introduced in calculated positions with the displacement factors of the host carbon atoms. Structure solution and refinement were performed using the SHELXL package [39].

### 3.3. Synthesis of Ligand ***L3H***

A solution of *n*-BuLi in hexane (1.6 M, 8.3 mL, 13.2 mmol) was added to a THF (20 mL) solution of 8-aminoquinoline (1.73 g, 12.0 mmol) at −78 °C under nitrogen atmosphere. The reaction was allowed to warm up to room temperature and stirred overnight. The solution was transferred into a THF (20 mL) solution of *ortho*-C_6_H_4_F(C(H)=N-4-OCH_3_C_6_H_4_) (2.29 g, 10.0 mmol) at room temperature and then heated at 50 °C for 8 h. After cooling to room temperature, the reaction mixture was distributed in CH_2_Cl_2_ and H_2_O. The aqueous layer was extracted with CH_2_Cl_2_ (15 mL × 2). The combined organic phase was dried over anhydrous MgSO_4_ and filtered. The solvent was removed in vacuo to give the crude product, which was recrystallized from methanol-hexane to afford **L3H** as a yellow solid. Yield: 2.12 g (60%). ^1^H NMR (400 MHz, CDCl_3_) δ 12.78 (s, 1H), 8.96 (d, J = 2.8 Hz, 1H), 8.74 (s, 1H), 8.13 (d, J = 8.2 Hz, 1H), 7.89 (d, J = 8.0 Hz, 2H), 7.52 (t, J = 6.7 Hz, 1H), 7.51–7.42 (m, 4H), 7.37 (dd, J = 17.3, 8.2 Hz, 2H), 6.96 (dd, J = 14.8, 8.1 Hz, 3H), 3.84 (s, 3H) ppm. ^13^C{^1^H} NMR (101 MHz, CDCl_3_) δ 159.1, 158.3, 148.2, 143.7, 143.6, 140.6, 139.2, 135.9, 134.5, 131.1, 129.2, 126.8, 122.4, 121.8, 121.5, 118.7, 118.6, 115.1, 114.3, 111.8, 55.4 ppm. MS (ESI, *m*/*z*): 354.2 [M + H]^+^.

### 3.4. Synthesis of Complex ***2***

A solution of *n*-BuLi (1.6 mol/L) in hexane (0.7 mL, 1.1 mmol) was added dropwise to a THF (20 mL) solution of ligand **L2H** (0.37 g, 1.0 mmol) at −78 °C under nitrogen atmosphere. After stirring for 1 h, CuCl_2_ (0.13 g, 1.0 mmol) was added in one portion. The mixture was gradually warmed to room temperature and stirred overnight. Evaporation of the solvent in vacuo gave the crude product, which was extracted with 15 mL of CH_2_Cl_2_ and then filtered through Celite. The solution was evaporated under reduced pressure to a volume of ~3 mL. The resulting residue was recrystallized from CH_2_Cl_2_/hexane to give complex **2** as a brown powder. Yield: 0.30 g (65%). Single crystals for X-ray analysis were grown from CH_2_Cl_2_/hexane at room temperature. M.p. 229–230 °C. IR (KBr disk, cm^−1^): 2899 (w), 1613 (s), 1598 (s), 1568 (m), 1502 (m), 1456 (m), 1437 (m), 1377 (m), 1326 (m), 1188 (s), 1160 (s), 849 (m), 824 (m), 761 (m), 744 (m). HR-MS (ESI-TOF): calcd for C_25_H_22_ClCuN_3_, [M-Cl]^+^ 427.1104, found 427.1101.

### 3.5. Synthesis of Complex ***3***

Following the procedure as described for complex **1** using **L3H** as the ligand, complex **3** was obtained as a brown solid. Yield: 0.31 g (68%). M.p. 211–212 °C. IR (KBr disk, cm^−1^): 2911 (w), 1612 (s), 1597 (s), 1570 (m), 1507 (s), 1500 (s), 1470 (s), 1440 (s), 1379 (s), 1333 (m), 1245 (m), 1180 (m), 1164 (s), 828 (m), 803 (w), 763 (m), 741 (m). HR-MS (ESI-TOF): calcd for C_23_H_18_ClCuN_3_O, [M-Cl]^+^ 415.0740, found 415.0748.

### 3.6. Synthesis of Complex ***4***

Following the procedure as described for complex **1** using **L4H** as the ligand, complex **4** was obtained as a brown solid. Yield: 0.31 g (61%). M.p. 252–253 °C. IR (KBr disk, cm^−1^): 2924 (w), 1603 (s), 1571 (m), 1535 (m), 1501 (m), 1458 (s), 1436 (s), 1383 (m), 1224 (w), 1183 (m), 1157 (s), 831 (m), 818 (m), 760 (m), 746 (m). HR-MS (ESI-TOF): calcd for C_22_H_15_BrClCuN_3_, [M-Cl]^+^ 462.9740, found 462.9738.

### 3.7. Synthesis of Complex ***5***

Following the procedure as described for complex **1** using **L5H** as the ligand, complex **5** was obtained as a brown solid. Yield: 0.25 g (55%). M.p. 266–267 °C. IR (KBr disk, cm^−1^): 2923 (w), 1602 (m), 1570 (m), 1536 (s), 1500 (m), 1457 (m), 1436 (m), 1386 (m), 1222 (w), 1180 (m), 1159 (m), 830 (w), 822 (w), 750 (w), 739 (w). HR-MS (ESI-TOF): calcd for C_22_H_15_Cl_2_CuN_3_, [M-Cl]^+^ 419.0245, found 419.0242. 

### 3.8. General Procedure for the Cu-Catalyzed Chan-Lam Coupling Reactions

A mixture of benzimidazoles **6** (0.20 mmol), arylboronic acid **7** (0.60 mmol), Cu catalyst **3** (3 mol%) and CH_3_OH (1.0 mL) were added in a 10 mL reaction tube. The reaction mixture was then heated at 40 °C in an oil bath for 12 h under the air atmosphere. After the reaction was completed, the reaction was cooled to room temperature and the volatiles were removed in vacuo. The resulting residue was purified by column chromatography on Al_2_O_3_ using CH_2_Cl_2_ and petroleum ether as eluent to give the product **8**, which was further identified and confirmed by NMR (Figures S6–S54).

## 4. Conclusions

In summary, we have successfully synthesized and characterized a range of copper complexes containing amine-imine ligands. These Cu(II) complexes have proven to be effective catalysts in the Chan–Lam coupling reaction, facilitating the formation of C-N bonds without the need for a base. This catalytic system offers mild reaction conditions, ease of operation, and excellent compatibility with various functional groups. Copper amine-imine complexes of this nature hold the promise of broadening the scope of efficient C-N bond formation. Ongoing research in our laboratory is exploring further applications for these catalysts.

## Figures and Tables

**Table 1:** Summary of the crystal data for complexes **2** and **4**.

Complex	2	4
Formula	C_25_H_22_ClCuN_3_	C_22_H_15_BrClCuN_3_
*F*_w_	463.44	500.27
*T* (K)	199.99 (10)	199.99 (10)
Crystal system	monoclinic	monoclinic
Space group	*C*c	*P*2_1_/*c*
*a* (Å)	17.2718 (8)	15.5617 (4)
*b* (Å)	15.2065 (6)	9.4245 (2)
*c* (Å)	8.1558 (3)	27.3576 (7)
*α* (°)	90	90
*β* (°)	98.532 (4)	96.439 (2)
*γ* (°)	90	90
Volume (Å^3^)	2118.36 (15)	3986.99 (17)
*Z*	4	8
*D_calc_* (g/cm^3^)	1.453	1.667
*μ* (mm^−1^)	2.736	5.220
*F* (000)	956.0	1992.0
Crystal size (mm)	0.14 × 0.12 × 0.1	0.14 × 0.12 × 0.1
2Θ range for data collection (°)	7.784 to 146.896	5.716 to 147.15
Reflections collected	4242	14,755
Data/restraints/parameters	2723/2/274	7793/0/505
Goodness-of-fit on *F*^2^	1.074	1.008
*R*_1_, w*R*_2_ [*I* > 2*σ*(*I*)]	0.0327, 0.0888	0.0533, 0.1325
*R*_1_, w*R*_2_ (all data)	0.0339, 0.0899	0.0758, 0.1496
Max. peak (e·Å^−3^)	0.49	0.58
Mini. peak (e·Å^−3^)	−0.71	−0.71
CCDC	2248465	2248467

**Table 2:**
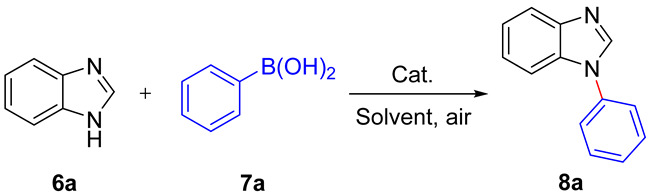
Optimization of Chan–Lam coupling reaction *^a^*.

Entry	Cat. (mol%)	Solvent	T (°C)	Time (h)	Yield (%) *^b^*
1	**1** (5)	MeOH	50	20	90
2	**1** (5)	*^i^*PrOH	50	20	6
3	**1** (5)	1,4-Dioxane	50	20	40
4	**1** (5)	DMF	50	20	trace
5	**1** (5)	CH_3_CN	50	20	trace
6	**1** (5)	THF	50	20	46
7	**1** (5)	Toluene	50	20	25
8	**1** (5)	MeOH	50	16	92
9	**1** (5)	MeOH	50	12	90
10	**1** (5)	MeOH	50	8	83
11	**1** (5)	MeOH	40	12	92
12	**1** (5)	MeOH	rt	12	81
13	**1** (5)	MeOH	60	12	88
14	**1** (3)	MeOH	40	12	93
15	**1** (2)	MeOH	40	12	84
16	**1** (1)	MeOH	40	12	71
17	**2** (3)	MeOH	40	12	91
**18**	**3** (**3**)	**MeOH**	**40**	**12**	**96**
19	**4** (3)	MeOH	40	12	94
20	**5** (3)	MeOH	40	12	90
21	CuCl_2_ (3)	MeOH	40	12	48
22	-	MeOH	40	12	-
23 *^c^*	**3** (3)	MeOH	40	12	-

*^a^* Reaction conditions: **6a** (0.20 mmol), **7a** (0.60 mmol), **Cat**. (3 mol%), MeOH (1.0 mL), 40 °C, 12 h, under air. *^b^* Isolated yield. *^c^* Under an N_2_ atmosphere.

**Table 3:** Substrate scope of Chan–Lam coupling reaction catalyzed by **3** *^a^*.

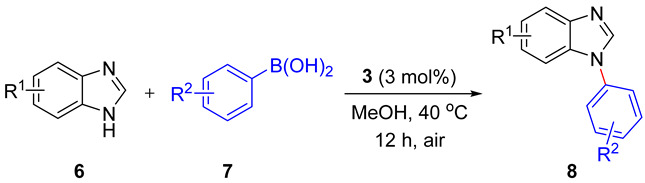			
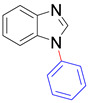	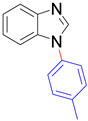	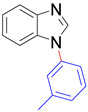	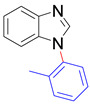
**8a**, 96%	**8b**, 96%	**8c**, 95%	**8d**, 91%
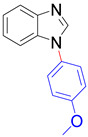	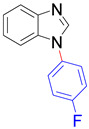	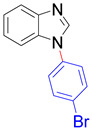	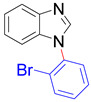
**8e**, 89%	**8f**, 94%	**8g**, 93%	**8h**, 85%
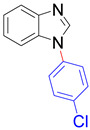	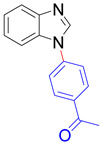	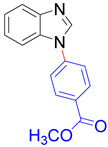	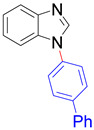
**8i**, 96%	**8j**, 88%	**8k**, 91%	**8l**, 83%
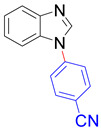	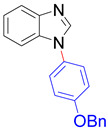	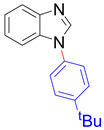	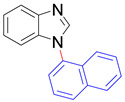
**8m**, 75%	**8n**, 77%	**8o**, 84%	**8p**, 87%
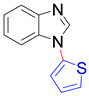	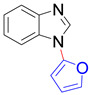	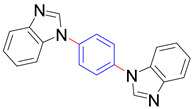	
**8q**, trace	**8r**, trace	**8s***^ b^*, 76%	
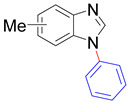	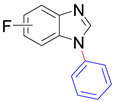	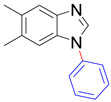	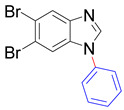
**8t**/**8t’** (5/6-Me), 95%	**8u**/**8u’** (5/6-F), 79%	**8v**, 93%	**8w**, 58%
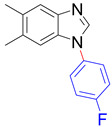	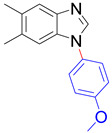		
**8x**, 88%	**8y**, 90%		

*^a^* Reaction conditions: **6** (0.20 mmol), **7** (0.60 mmol), **3** (3 mol%), MeOH (1.0 mL), 40 °C, 12 h, under air, isolated yield. *^b^* benzimidazole **6a** (0.20 mmol), 1,4-phenylenediboronic acid **7s** (0.20 mmol).

## Data Availability

Not applicable.

## Supplementary Materials

The following supporting information can be downloaded at: https://www.mdpi.com/article/10.3390/molecules28217406/s1, Figures S1–S4: HR-MS spectra of copper complexes **2**-**5**; Table S1 and Figure S5: the EPR spectra and g values of copper complexes **2**-**5**; Figures S6–S54: NMR spectra of catalytic products.
