# Supplementary material for: Copper Complexes with N,N,N-Tridentate Quinolinyl Anilido-Imine Ligands: Synthesis and Their Catalytic Application in Chan−Lam Reactions
Source: Molecules. 2023 Nov 3;28(21):7406. doi: 10.3390/molecules28217406 (PMC10647278; doi:10.3390/molecules28217406)
Supplement: Supplementary file 1 [file molecules-28-07406-s001.zip › molecules-2663391-supplementary.pdf]

Supporting Information

Copper Complexes with N,N,N-Tridentate Quinolinyl Anilido-Imine Ligand: Synthesis and Their Catalytic Application in Chan−Lam Reactions

Xiaoyu Zhou [1], Jiaxin Yang [1], Guang Zeng [2], Zhiqiang Hao [1,*], Zhan-Gang Han [1], Jin Lin [1,*] and Guo-Liang Lu [3,*]

1 National Experimental Chemistry Teaching Center (Hebei Normal University), Hebei Key Laboratory of Organic Functional Molecules, College of Chemistry and Materials Science, Hebei Normal University, Shi-jiazhuang 050024, China.

2 State Key Laboratory of Catalysis, Dalian Institute of Chemical and Physics, Chinese Academy of Sciences, Zhongshan Road 457, Dalian 116023, China.

3 Auckland Cancer Society Research Centre, Faculty of Medical and Health Sciences and Maurice Wilkins Centre, The University of Auckland, Auckland 1142, New Zealand.

* Correspondence: zqhao@hebtu.edu.cn; linjin@hebtu.edu.cn; gl.lu@auckland.ac.nz

Table of contents

1. HR-MS spectra of copper complexes **2-5**……………………………S2

2. EPR spectra and the g values of copper complexes **2-5**……….……….S4

3. NMR data of catalytic products…………………..…………………..S5

4. NMR spectra of catalytic products………………..…………………..S14

1. HR-MS spectra of copper complexes 2-5

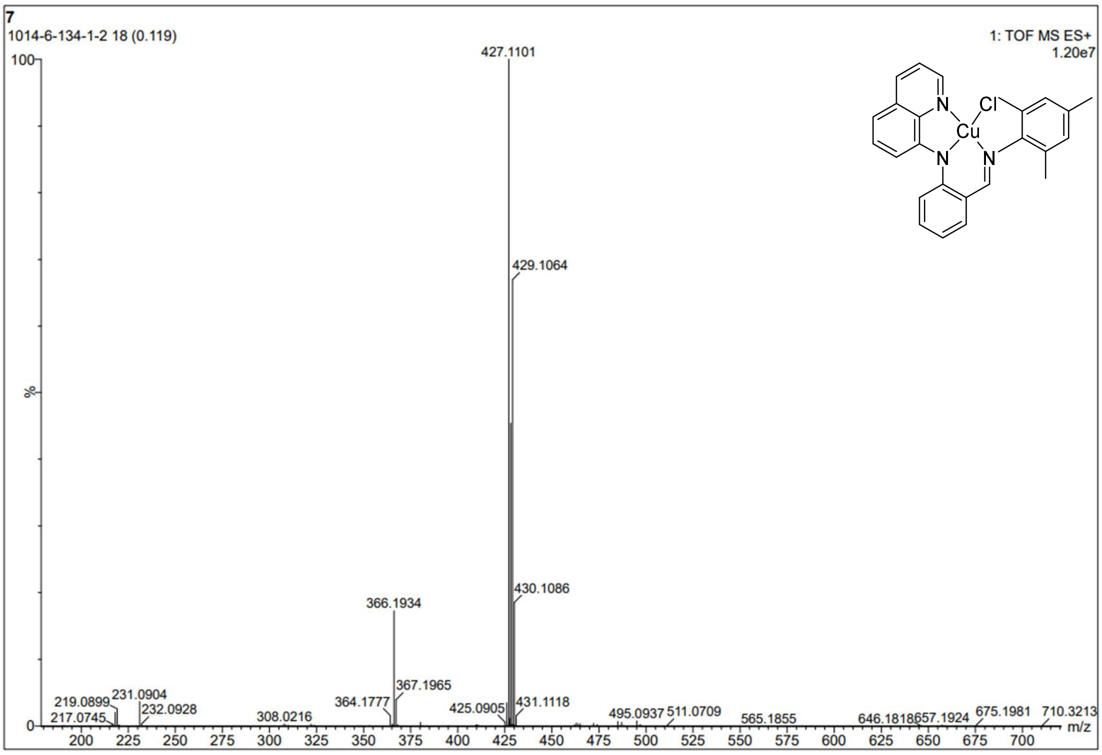

Figure S1. HR-MS spectra of **2**

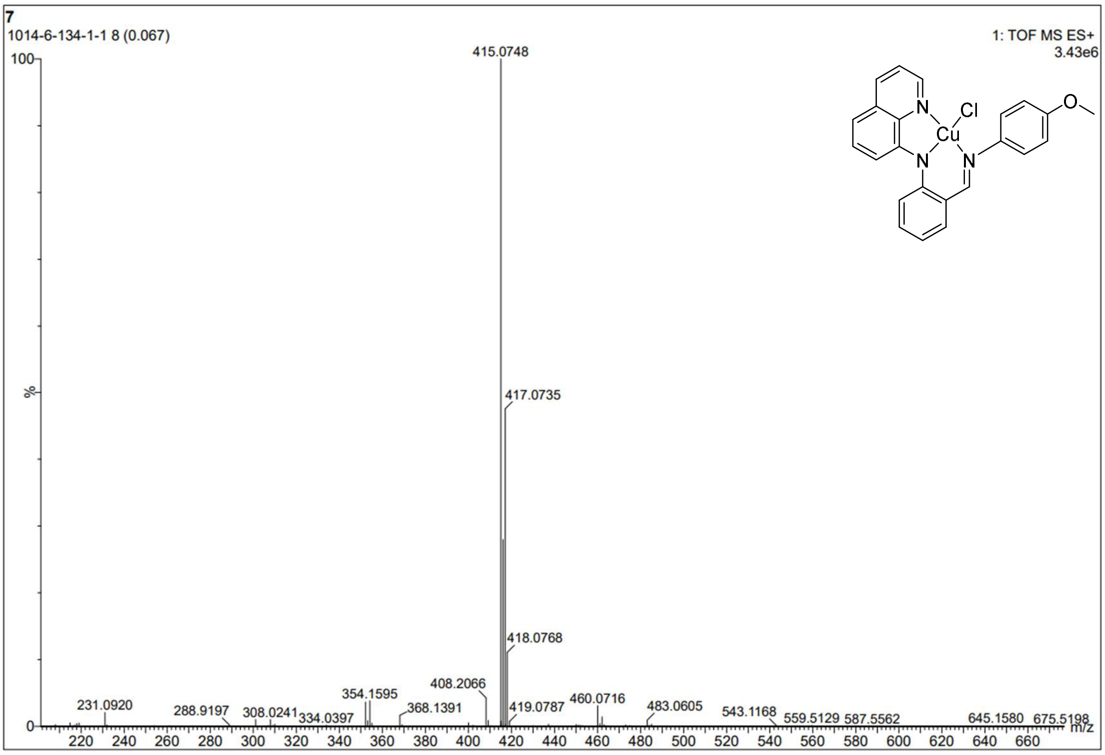

Figure S2. HR-MS spectra of **3**

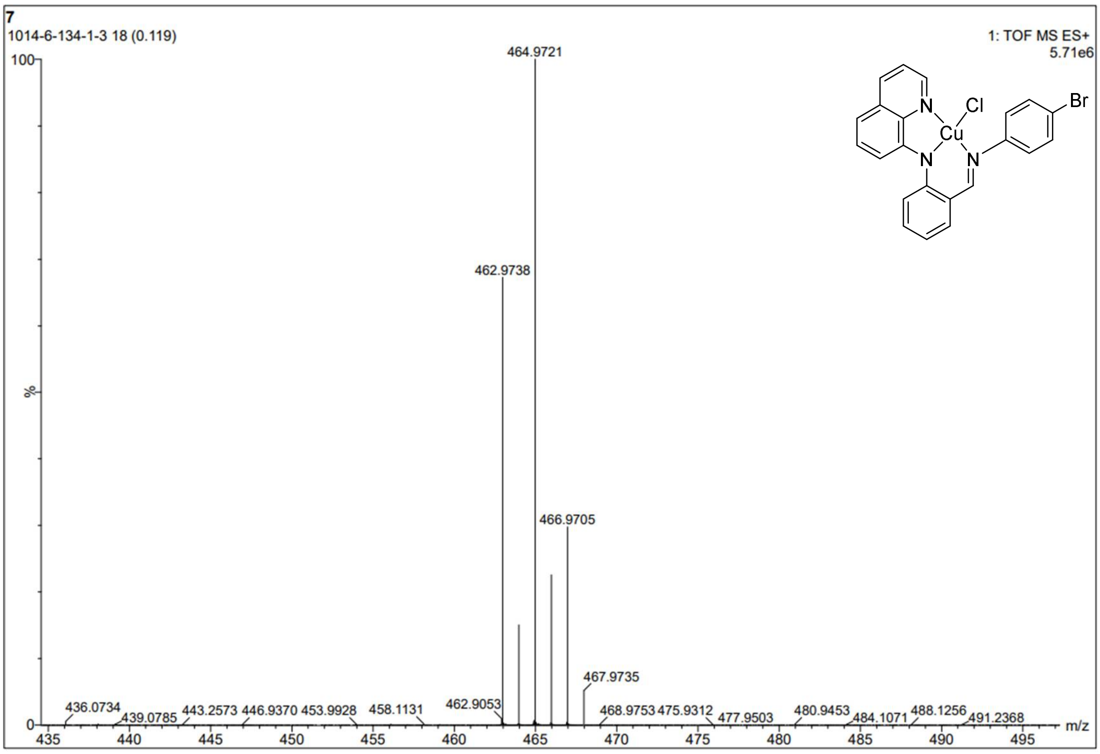

Figure S3. HR-MS spectra of **4**

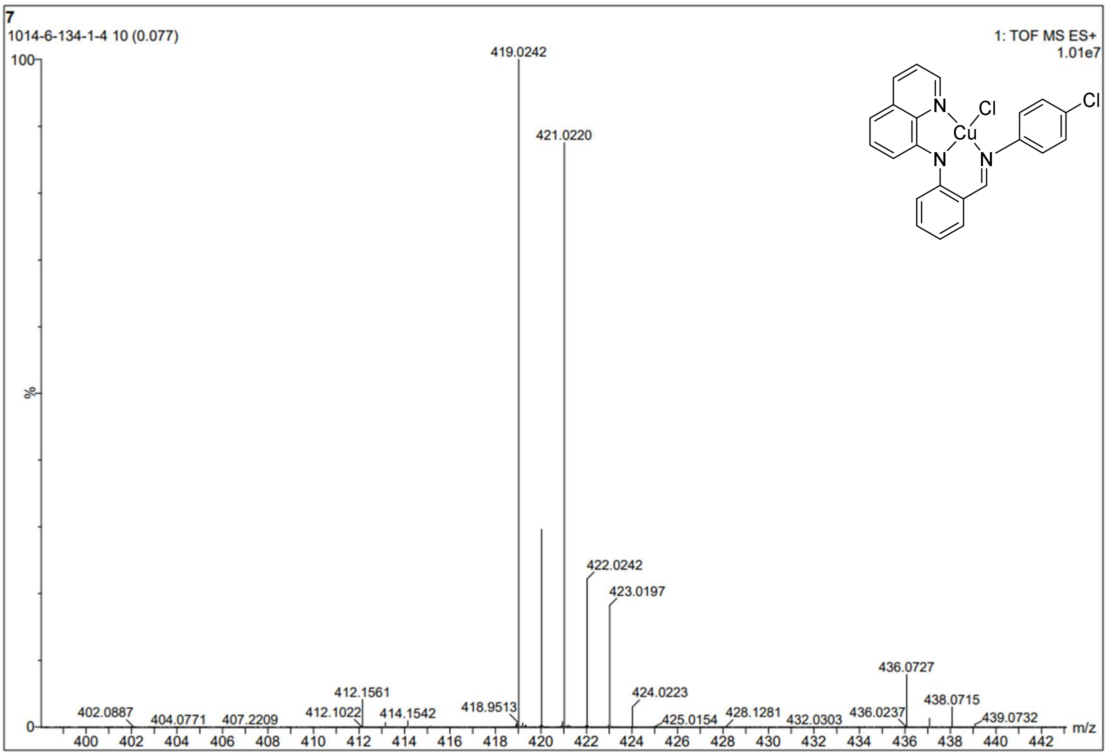

Figure S4. HR-MS spectra of **5**

2. EPR spectra and the g values of copper complexes 2-5

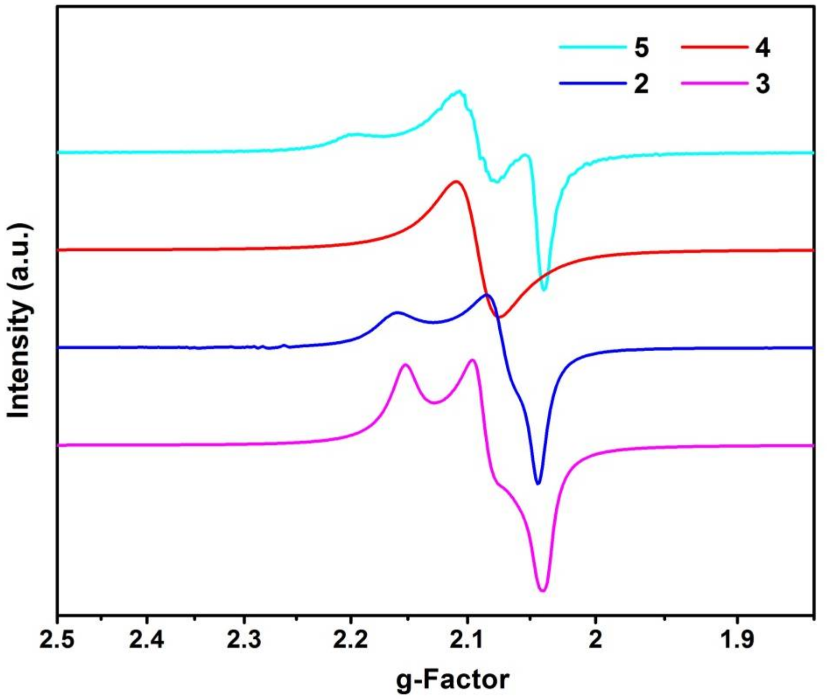

Figure S5. The g values of copper complexes **2-5**

Table S1 The ESR parameters for the Cu^{II} complexes **2-5**

Cu Complex	g_x	g_y	g_z	g_{iso}
2	2.04	2.08	2.16	—
3	2.04	2.09	2.15	—
4	—	—	—	2.09
5	2.04	2.05	2.11	—

3. NMR data of catalytic products

3.1 8a

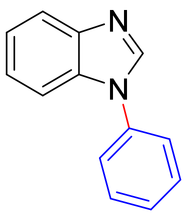

^{1}H NMR (400 MHz, $CDCl_3$) δ 8.17 (s, 1H), 7.89 (d, J = 8.8 Hz, 1H), 7.61 – 7.49 (m, 5H), 7.47 (t, J = 7.3 Hz, 1H), 7.38 – 7.30 (m, 2H) ppm.

$^{13}C\{^{1}H\}$ NMR (101 MHz, $CDCl_3$) δ 143.8, 142.2, 136.3, 133.6, 130.0, 128.0, 124.0, 123.7, 122.8, 120.5, 110.4 ppm.

3.2 8b

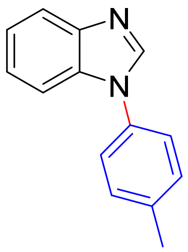

^{1}H NMR (400 MHz, $CDCl_3$) δ 8.07 (s, 1H), 7.88 (d, J = 7.7 Hz, 1H), 7.50 (d, J = 6.3 Hz, 1H), 7.36 (t, J = 10.6 Hz, 5H), 7.32 – 7.28 (m, 1H), 2.44 (s, 3H) ppm.

$^{13}C\{^{1}H\}$ NMR (101 MHz, $CDCl_3$) δ 143.9, 142.3, 138.0, 133.7, 133.7, 130.4, 123.8, 123.4, 122.5, 120.4, 110.4, 21.0 ppm.

3.3 8c

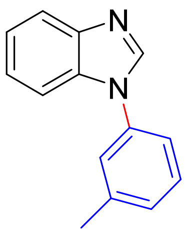

^{1}H NMR (400 MHz, $CDCl_3$) δ 8.09 (s, 1H), 7.87 (dd, J = 5.4, 2.8 Hz, 1H), 7.55 – 7.50 (m, 1H), 7.43 (t, J = 7.6 Hz, 1H), 7.36 – 7.23 (m, 5H), 2.45 (s, 3H) ppm.

$^{13}C\{^{1}H\}$ NMR (101 MHz, $CDCl_3$) δ 143.9, 142.3, 140.2, 136.2, 133.7, 129.8, 128.7, 124.6, 123.6, 122.7, 121.0, 120.5, 110.5, 21.4 ppm.

3.4 8d

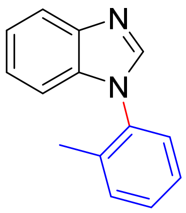

^{1}H NMR (400 MHz, $CDCl_3$) δ 7.97 (s, 1H), 7.89 (d, *J* = 7.8 Hz, 1H), 7.42 (s, 2H), 7.39 – 7.27 (m, 4H), 7.13 (d, *J* = 7.8 Hz, 1H), 2.10 (s, 3H) ppm.

$^{13}C\{^1H\}$ NMR (101 MHz, $CDCl_3$) δ 143.2, 142.9, 135.3, 134.7, 131.5, 129.3, 128.8, 127.6, 127.1, 123.5, 122.4, 120.3, 110.4, 17.5 ppm.

3.5 8e

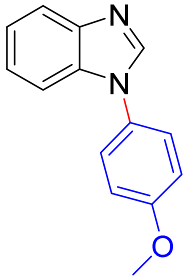

^{1}H NMR (400 MHz, $CDCl_3$) δ 8.05 (s, 1H), 7.87 (d, *J* = 7.2 Hz, 1H), 7.44 (t, *J* = 7.0 Hz, 1H), 7.42 – 7.37 (m, 2H), 7.35 – 7.28 (m, 2H), 7.10 – 7.02 (m, 2H), 3.88 (s, 3H) ppm.

$^{13}C\{^1H\}$ NMR (101 MHz, $CDCl_3$) δ 159.3, 143.7, 142.5, 134.2, 129.1, 125.7, 123.5, 122.6, 120.4, 115.1, 110.3, 55.6 ppm.

3.6 8f

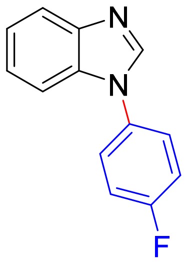

^{1}H NMR (400 MHz, $CDCl_3$) δ 8.06 (s, 1H), 7.90 – 7.85 (m, 1H), 7.47 (dt, *J* = 9.1, 4.6 Hz, 3H), 7.36 – 7.30 (m, 2H), 7.26 (t, *J* = 8.4 Hz, 2H) ppm.

$^{13}C\{^1H\}$ NMR (101 MHz, $CDCl_3$) δ 163.1, 143.8, 142.2, 126.0, 125.9, 123.8,

122.8, 120.6, 117.1, 116.8, 110.1 ppm.

^{19}F NMR (376 MHz, $CDCl_3$) δ -112.8 ppm.

3.7 8g

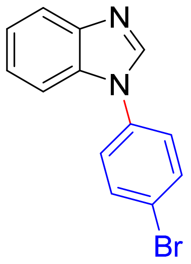

^{1}H NMR (400 MHz, $CDCl_3$) δ 8.06 (s, 1H), 7.87 (dd, *J* = 4.9, 3.9 Hz, 1H), 7.67 (d, *J* = 8.5 Hz, 2H), 7.50 – 7.44 (m, 1H), 7.41 – 7.29 (m, 4H) ppm.

^{13}C{^{1}H} NMR (101 MHz, $CDCl_3$) δ 143.9, 141.8, 135.2, 133.3, 133.1, 125.4, 123.8, 122.9, 121.5, 120.6, 110.1 ppm.

3.8 8h

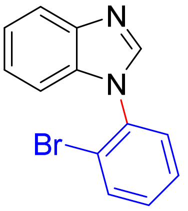

^{1}H NMR (400 MHz, $CDCl_3$) δ 8.03 (s, 1H), 7.88 (d, *J* = 7.6 Hz, 1H), 7.80 (d, *J* = 8.0 Hz, 1H), 7.49 (t, *J* = 7.5 Hz, 1H), 7.39 (ddd, *J* = 12.3, 6.4, 5.0 Hz, 2H), 7.35 – 7.26 (m, 2H), 7.18 (d, *J* = 7.9 Hz, 1H) ppm.

^{13}C{^{1}H} NMR (101 MHz, $CDCl_3$) δ 143.1, 142.8, 135.0, 134.2, 134.1, 130.5, 129.0, 128.6, 123.6, 122.6, 121.3, 120.3, 110.4 ppm.

3.9 8i

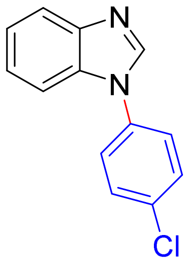

^{1}H NMR (400 MHz, $CDCl_3$) δ 8.06 (s, 1H), 7.87 (dd, *J* = 4.4, 2.9 Hz, 1H), 7.56 –

7.49 (m, 2H), 7.49 – 7.45 (m, 1H), 7.42 (dd, J = 8.6, 2.4 Hz, 2H), 7.35 – 7.29 (m, 2H) ppm.

$^{13}C\{^{1}H\}$ NMR (101 MHz, $CDCl_3$) δ 143.9, 141.9, 134.7, 133.7, 133.4, 130.1, 125.1, 123.8, 122.9, 120.6, 110.1 ppm.

3.10 8j

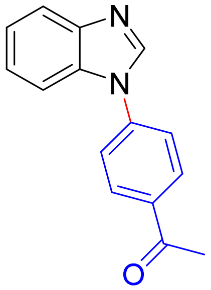

^{1}H NMR (400 MHz, $CDCl_3$) δ 8.14 (d, J = 2.5 Hz, 3H), 7.95 – 7.77 (m, 1H), 7.66 – 7.49 (m, 3H), 7.38 – 7.28 (m, 2H), 2.64 (s, 3H) ppm.

$^{13}C\{^{1}H\}$ NMR (101 MHz, $CDCl_3$) δ 196.5, 144.1, 141.7, 140.0, 136.0, 132.9, 130.2, 124.0, 123.2, 120.7, 110.3, 26.6 ppm.

3.11 8k

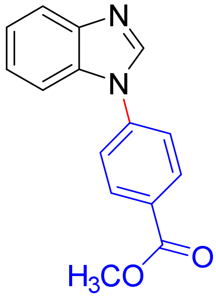

^{1}H NMR (400 MHz, $CDCl_3$) δ 8.23 (d, J = 8.4 Hz, 2H), 8.15 (s, 1H), 7.87 (dd, J = 6.2, 2.7 Hz, 1H), 7.57 (dd, J = 12.2, 5.9 Hz, 3H), 7.34 (dd, J = 5.9, 3.2 Hz, 2H), 3.95 (s, 3H) ppm.

$^{13}C\{^{1}H\}$ NMR (101 MHz, $CDCl_3$) δ 165.9, 144.2, 141.8, 140.1, 133.0, 131.5, 129.3, 124.0, 123.1, 120.8, 110.4, 52.3 ppm.

3.12 8l

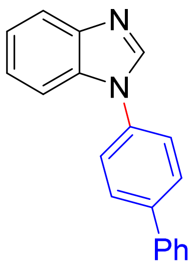

^{1}H NMR (400 MHz, $CDCl_3$) δ 8.16 (s, 1H), 7.94 – 7.90 (m, 1H), 7.78 (d, *J* = 8.4 Hz, 2H), 7.65 (d, *J* = 7.4 Hz, 2H), 7.62 – 7.55 (m, 3H), 7.50 (t, *J* = 7.5 Hz, 2H), 7.42 (t, *J* = 7.4 Hz, 1H), 7.38 – 7.34 (m, 2H) ppm.

^{13}C{^{1}H} NMR (101 MHz, $CDCl_3$) δ 144.2, 142.3, 141.1, 139.8, 135.5, 133.8, 129.1, 128.7, 128.0, 127.2, 124.3, 123.8, 122.9, 120.7, 110.6 ppm.

3.13 8m

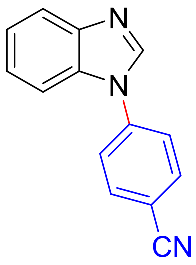

^{1}H NMR (400 MHz, $CDCl_3$) δ 8.15 (s, 1H), 7.88 (d, *J* = 7.1 Hz, 3H), 7.67 (d, *J* = 7.1 Hz, 2H), 7.59 – 7.53 (m, 1H), 7.41 – 7.34 (m, 2H) ppm.

^{13}C{^{1}H} NMR (101 MHz, $CDCl_3$) δ 144.2, 141.5, 140.0, 134.1, 132.7, 124.4, 123.8, 123.5, 121.0, 117.8, 111.4, 110.2 ppm.

3.14 8n

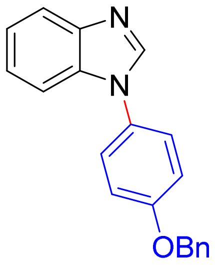

^{1}H NMR (400 MHz, $CDCl_3$) δ 8.05 (s, 1H), 7.89 (d, *J* = 7.9 Hz, 1H), 7.42 (ddd, *J* = 24.6, 10.5, 4.3 Hz, 8H), 7.35 – 7.30 (m, 2H), 7.14 (d, *J* = 8.6 Hz, 2H), 5.14 (s, 2H) ppm.

$^{13}C\{^1H\}$ NMR (101 MHz, $CDCl_3$) δ 158.3, 143.7, 142.4, 136.3, 134.1, 129.2, 128.6, 128.1, 127.4, 125.6, 123.4, 122.5, 120.4, 115.9, 110.3, 70.3 ppm.

3.15 8o

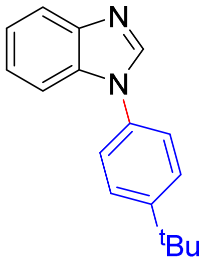

^1H NMR (400 MHz, $CDCl_3$) δ 8.10 (s, 1H), 7.88 (d, J = 8.5 Hz, 1H), 7.63 – 7.52 (m, 3H), 7.44 (d, J = 8.4 Hz, 2H), 7.37 – 7.30 (m, 2H), 1.40 (s, 9H) ppm.

$^{13}C\{^1H\}$ NMR (101 MHz, $CDCl_3$) δ 151.2, 143.9, 142.3, 133.7, 126.9, 123.6, 123.5, 122.6, 120.5, 110.5, 31.3 ppm.

3.16 8p

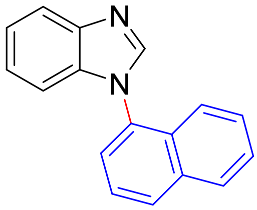

^1H NMR (400 MHz, $CDCl_3$) δ 8.12 (s, 1H), 8.04 – 7.91 (m, 3H), 7.56 (dt, J = 17.5, 7.4 Hz, 3H), 7.42 (d, J = 13.2 Hz, 2H), 7.34 (t, J = 7.6 Hz, 1H), 7.22 (t, J = 7.6 Hz, 1H), 7.07 (d, J = 8.0 Hz, 1H) ppm.

$^{13}C\{^1H\}$ NMR (101 MHz, $CDCl_3$) δ 143.7, 143.3, 135.6, 134.3, 132.2, 129.7, 129.6, 128.4, 127.4, 126.9, 125.4, 124.8, 123.5, 122.6, 122.4, 120.4, 110.7 ppm.

3.17 8s

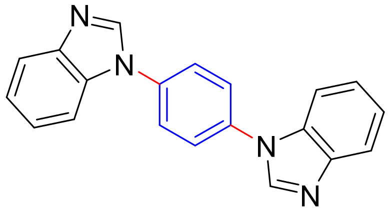

^1H NMR (400 MHz, $CDCl_3$) δ 8.20 (s, 2H), 7.96 – 7.90 (m, 2H), 7.77 (s, 4H), 7.64 – 7.59 (m, 2H), 7.40 (dd, J = 6.0, 3.1 Hz, 4H) ppm.

$^{13}C\{^1H\}$ NMR (101 MHz, $CDCl_3$) δ 144.1, 142.0, 135.9, 133.5, 128.7, 125.6, 124.1, 123.2, 120.9, 110.2 ppm.

3.18 8t/8t'

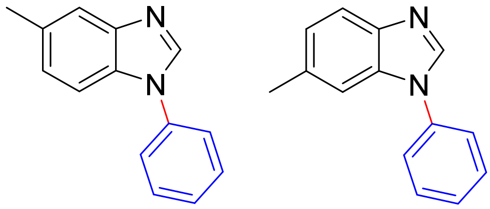

^1H NMR (400 MHz, $CDCl_3$) δ 8.05 (d, J = 10.2 Hz, 1H), 7.75 (d, J = 8.0 Hz, 0.5H), 7.66 (s, 0.5H), 7.54 (s, 2H), 7.51 – 7.39 (m, 4H), 7.32 (s, 0.5H), 7.15 (s, 1H), 2.49 (d, J = 11.6 Hz, 3H) ppm.

$^{13}C\{^1H\}$ NMR (101 MHz, $CDCl_3$) δ 144.3, 142.0, 141.7, 136.4, 136.3, 133.8, 133.6, 132.4, 131.6, 129.9, 127.8, 127.7, 125.1, 124.3, 123.9, 123.7, 120.2, 119.9, 110.2, 109.9, 21.7, 21.4 ppm.

3.19 8u/8u'

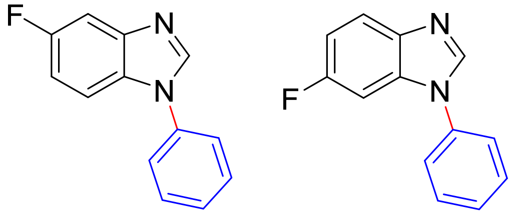

^1H NMR (400 MHz, $CDCl_3$) δ 8.10 (d, J = 11.9 Hz, 1H), 7.78 (dd, J = 8.8, 4.8 Hz, 0.5H), 7.62 – 7.39 (m, 6H), 7.19 (dd, J = 8.7, 1.9 Hz, 0.5H), 7.07 (t, J = 8.8 Hz, 1H) ppm.

$^{13}C\{^1H\}$ NMR (101 MHz, $CDCl_3$) δ 161.4, 160.6, 159.0, 158.3, 144.5, 143.5, 142.8, 140.3, 136.0, 135.9, 133.8, 133.7, 130.1, 130.1, 128.3, 128.2, 123.9, 123.8, 121.4, 121.2, 112.1, 111.9, 111.3, 111.1, 110.9, 110.8, 106.3, 106.1, 97.3, 97.0 ppm.

^{19}F NMR (376 MHz, $CDCl_3$) δ -117.5, -120.0 ppm.

3.20 8v

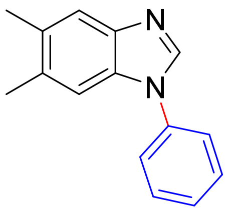

^{1}H NMR (400 MHz, $CDCl_3$) δ 8.00 (s, 1H), 7.64 (s, 1H), 7.54 (d, *J* = 6.8 Hz, 2H), 7.50 – 7.44 (m, 2H), 7.43 (s, 1H), 7.31 (s, 1H), 2.38 (d, *J* = 12.0 Hz, 6H) ppm.

$^{13}C\{^{1}H\}$ NMR (101 MHz, $CDCl_3$) δ 142.6, 141.4, 136.5, 132.8, 132.0, 131.5, 129.8, 127.6, 123.7, 120.4, 110.4, 20.5, 20.1 ppm.

3.21 8w

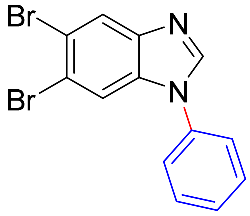

^{1}H NMR (400 MHz, $CDCl_3$) δ 8.14 (s, 1H), 8.08 (s, 1H), 7.80 (s, 1H), 7.60 (t, *J* = 7.6 Hz, 2H), 7.51 (t, *J* = 6.2 Hz, 1H), 7.47 (d, *J* = 7.4 Hz, 2H) ppm.

$^{13}C\{^{1}H\}$ NMR (101 MHz, $CDCl_3$) δ 144.3, 143.9, 135.4, 133.9, 130.3, 128.7, 125.0, 124.1, 119.2, 118.2, 115.0 ppm.

3.22 8x

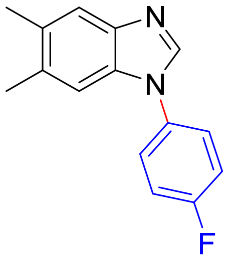

^{1}H NMR (400 MHz, $CDCl_3$) δ 7.95 (s, 1H), 7.62 (s, 1H), 7.51 – 7.38 (m, 2H), 7.25 (dd, *J* = 11.8, 5.6 Hz, 3H), 2.38 (d, *J* = 11.2 Hz, 6H) ppm.

$^{13}C\{^{1}H\}$ NMR (101 MHz, $CDCl_3$) δ 163.1, 160.6, 142.5, 141.5, 133.1, 132.7, 132.4, 131.9, 125.9, 120.6 , 117.1, 116.9, 110.3, 20.6, 20.3 ppm.

^{19}F NMR (376 MHz, $CDCl_3$) δ -113.2 ppm.

3.23 8y

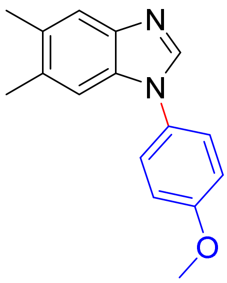

^{1}H NMR (400 MHz, $CDCl_3$) δ 7.93 (s, 1H), 7.62 (s, 1H), 7.38 (d, *J* = 8.6 Hz, 2H), 7.22 (s, 1H), 7.05 (d, *J* = 8.7 Hz, 2H), 3.87 (s, 3H), 2.37 (d, *J* = 14.2 Hz, 6H) ppm.

$^{13}C\{^1H\}$ NMR (101 MHz, $CDCl_3$) δ 159.0, 142.3, 141.6, 132.6, 131.4, 129.4, 125.4, 120.3, 114.9, 110.3, 55.5, 20.4, 20.2 ppm.

4. NMR spectra of catalytic products

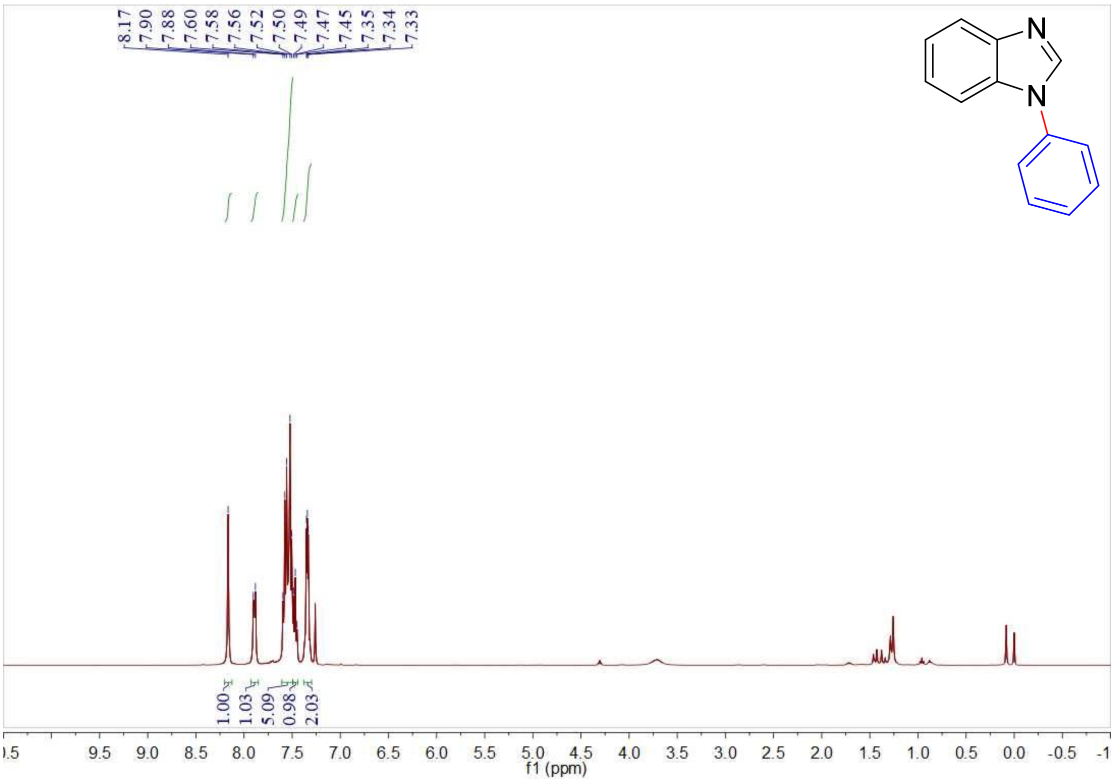

Figure S6. ^{1}H NMR spectra of **8a**

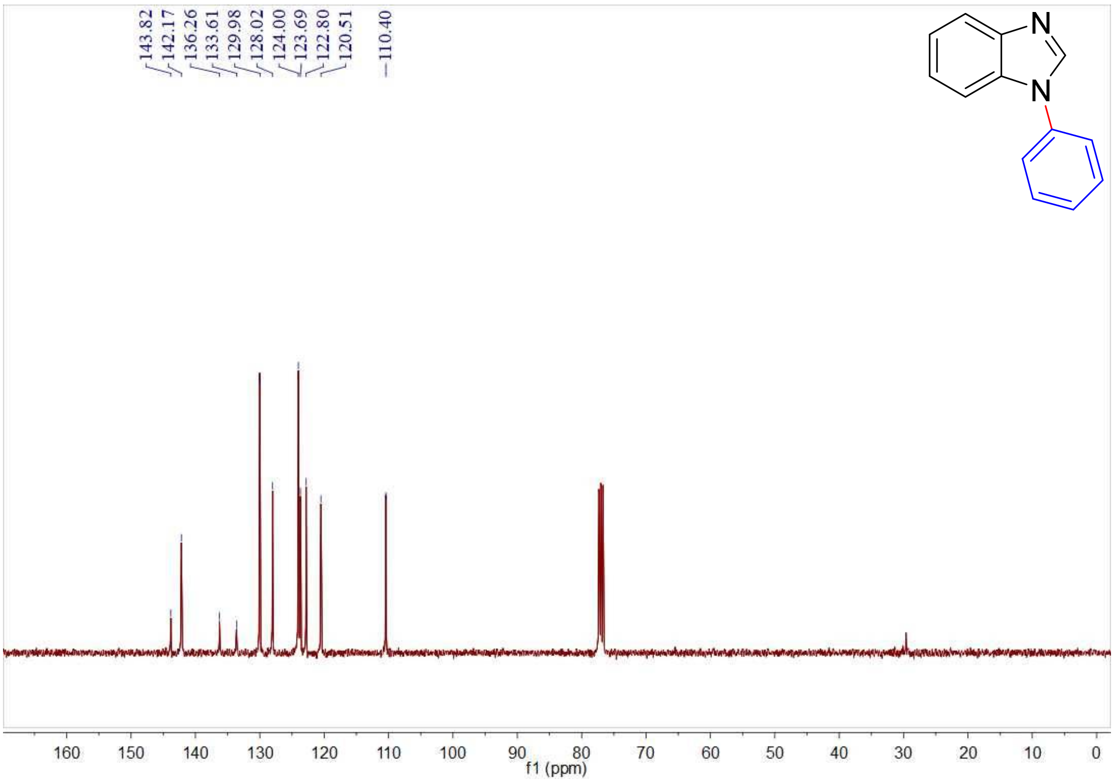

Figure S7. $^{13}C\{^{1}H\}$ NMR spectra of **8a**

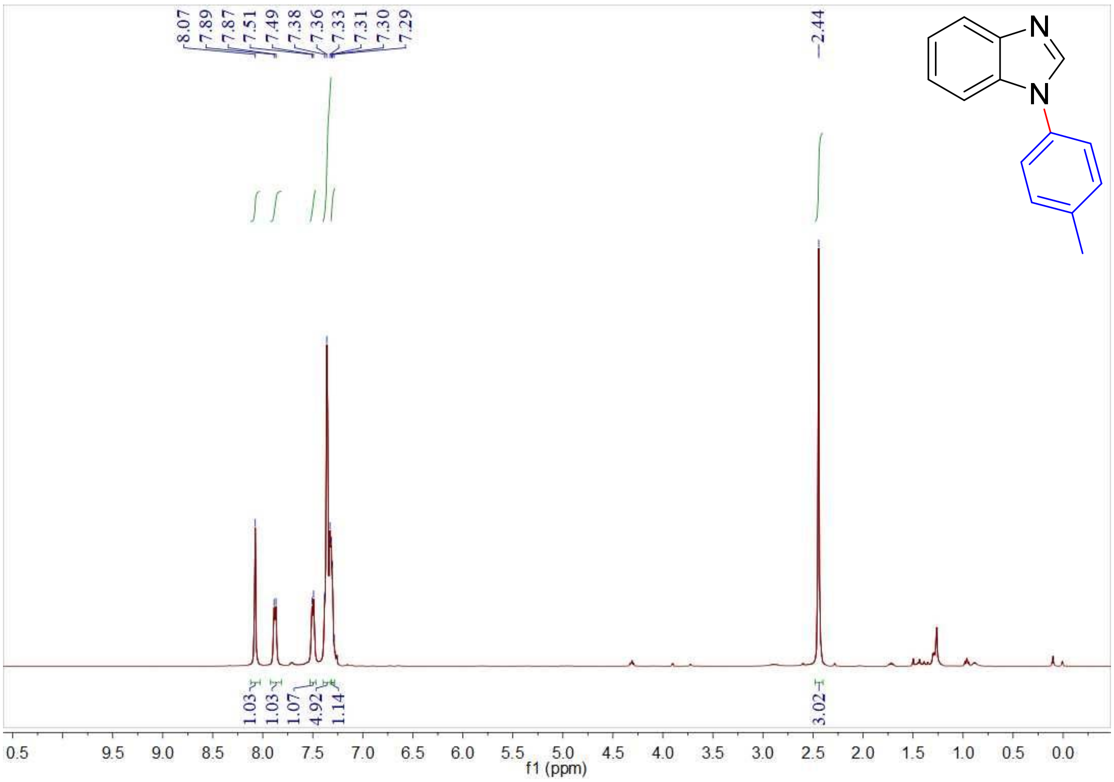

Figure S8. ^{1}H NMR spectra of **8b**

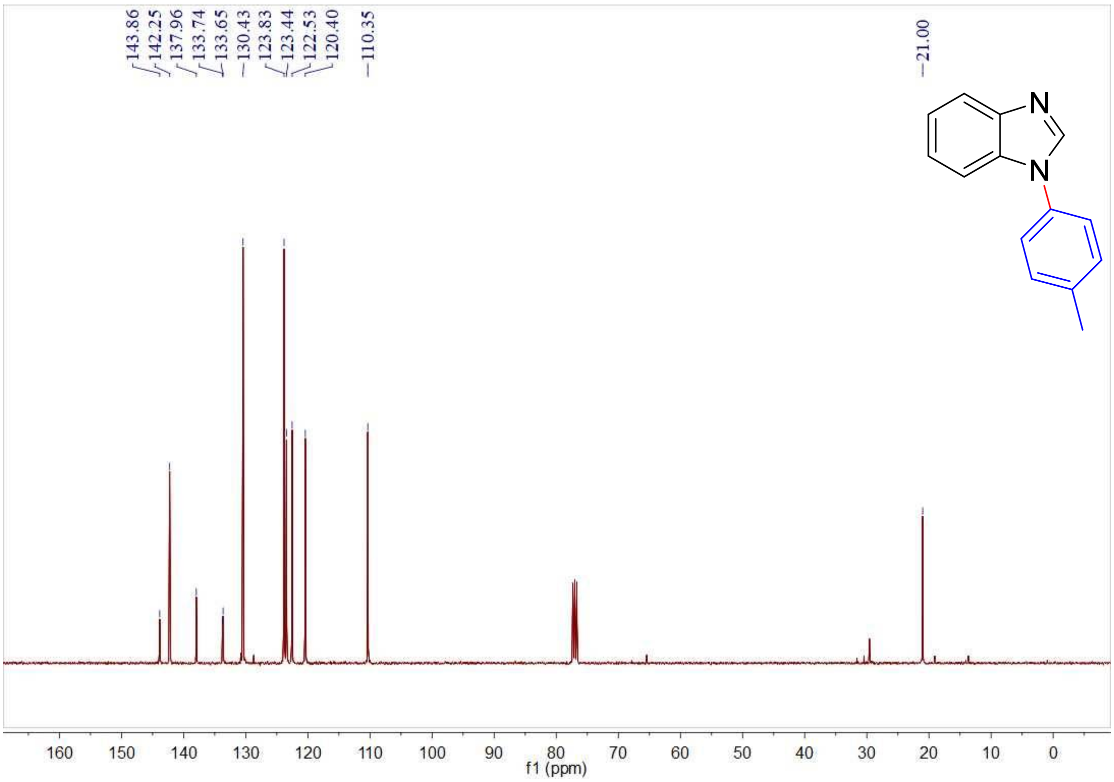

Figure S9. $^{13}C\{^{1}H\}$ NMR spectra of **8b**

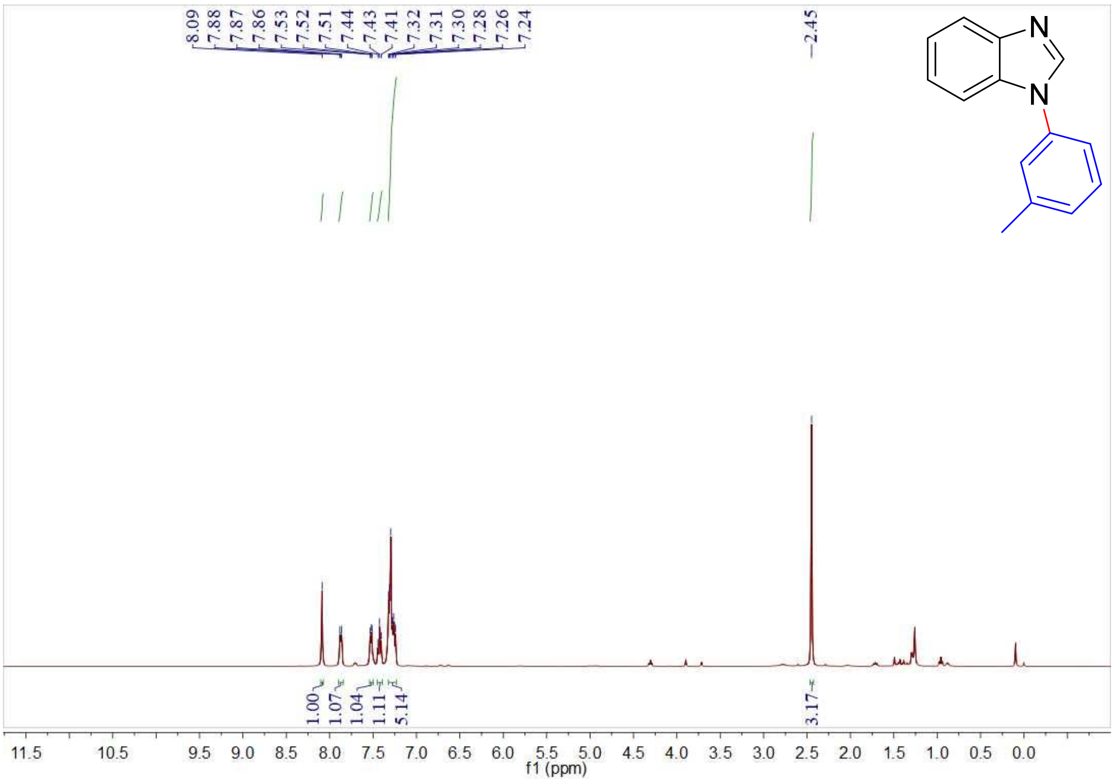

Figure S10. ^{1}H NMR spectra of **8c**

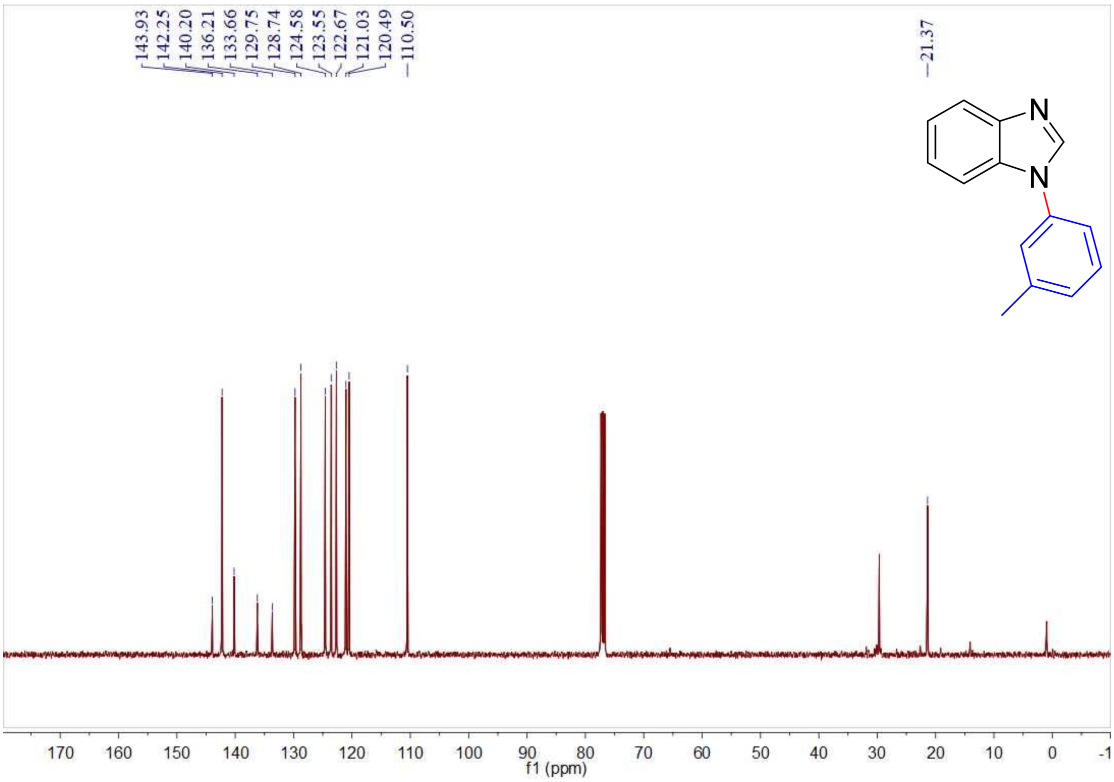

Figure S11. ^{13}C{^{1}H} NMR spectra of **8c**

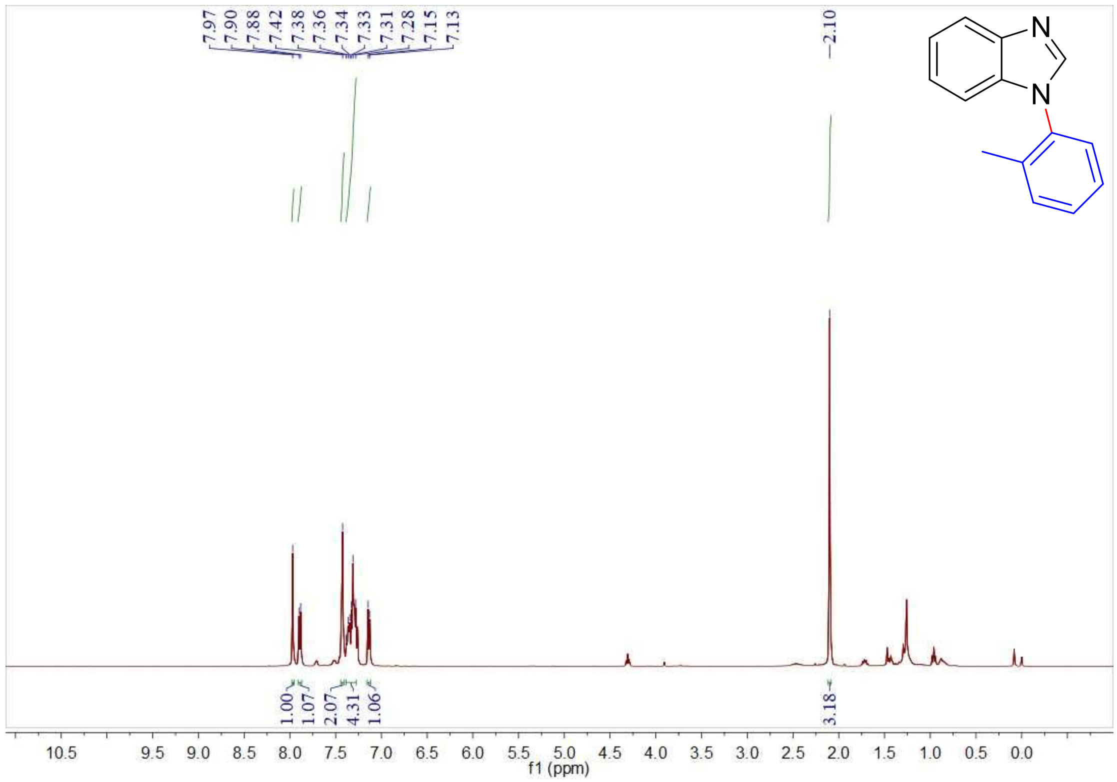

Figure S12. ^{1}H NMR spectra of **8d**

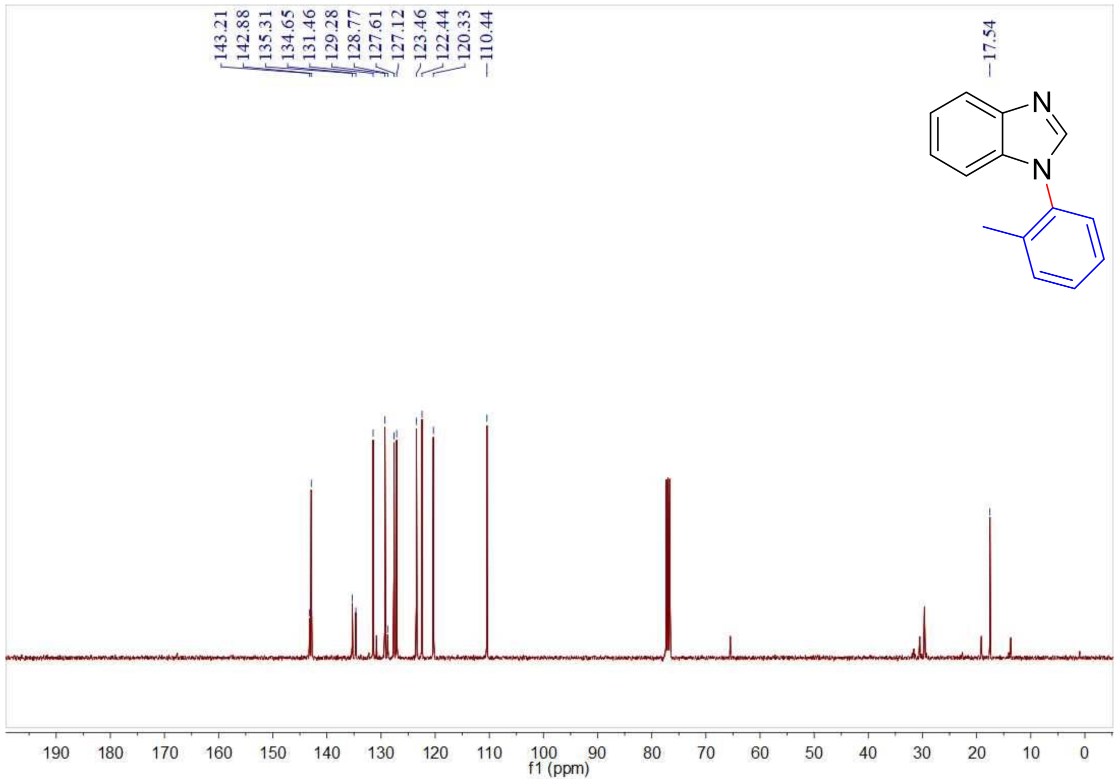

Figure S13. $^{13}C\{^{1}H\}$ NMR spectra of **8d**

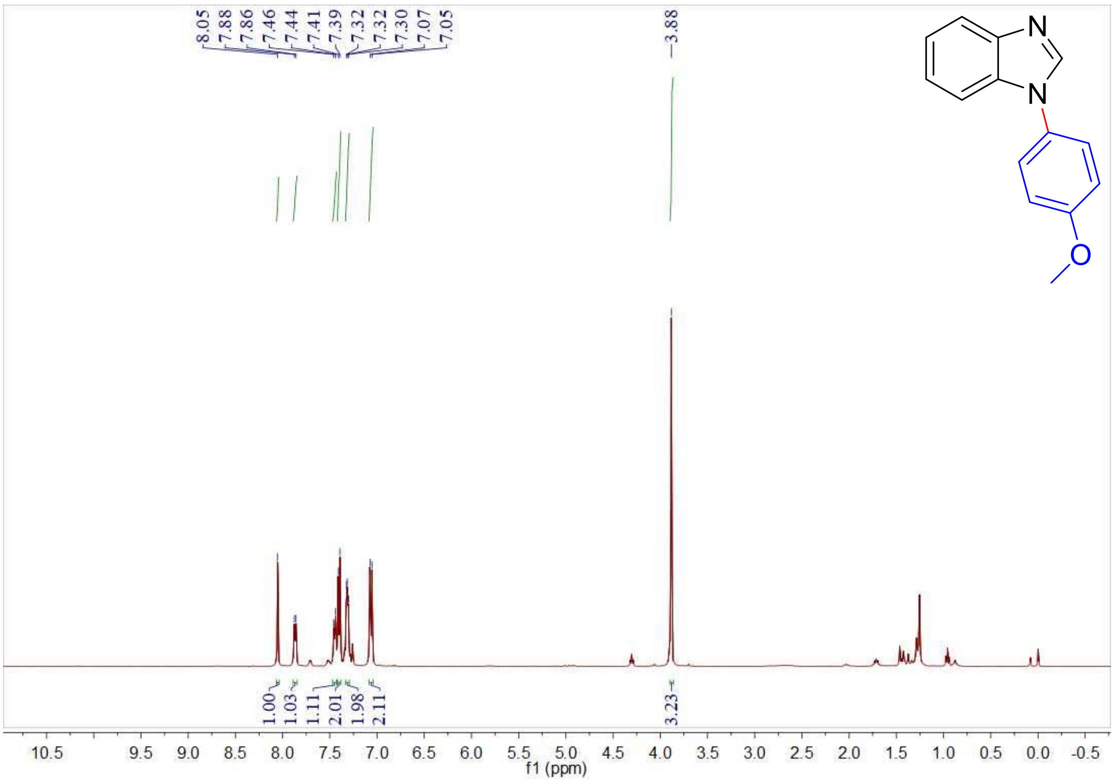

Figure S14. ^{1}H NMR spectra of **8e**

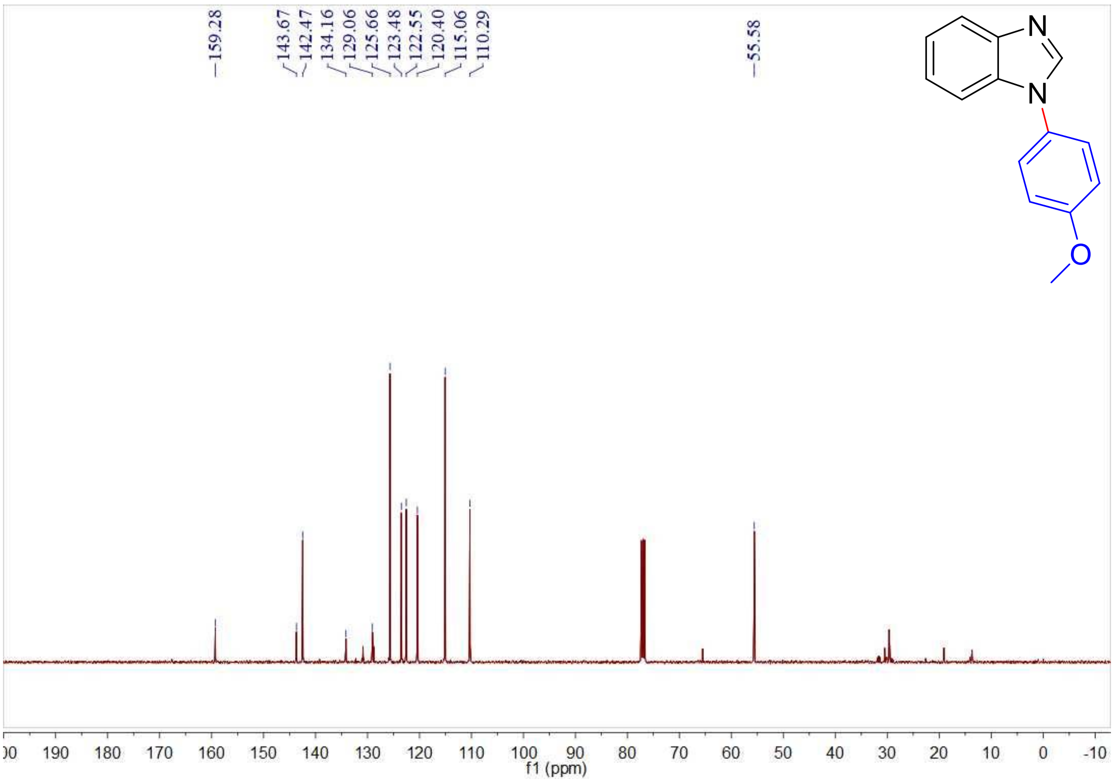

Figure S15. $^{13}C\{^{1}H\}$ NMR spectra of **8e**

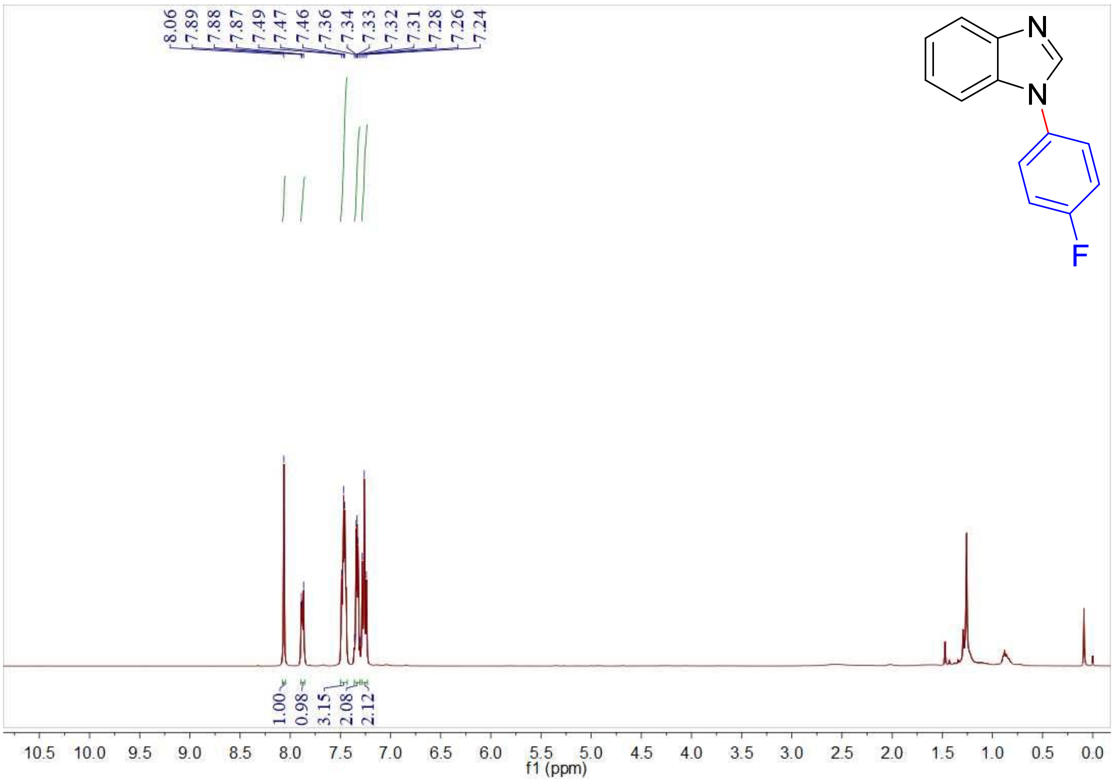

Figure S16. ^{1}H NMR spectra of **8f**

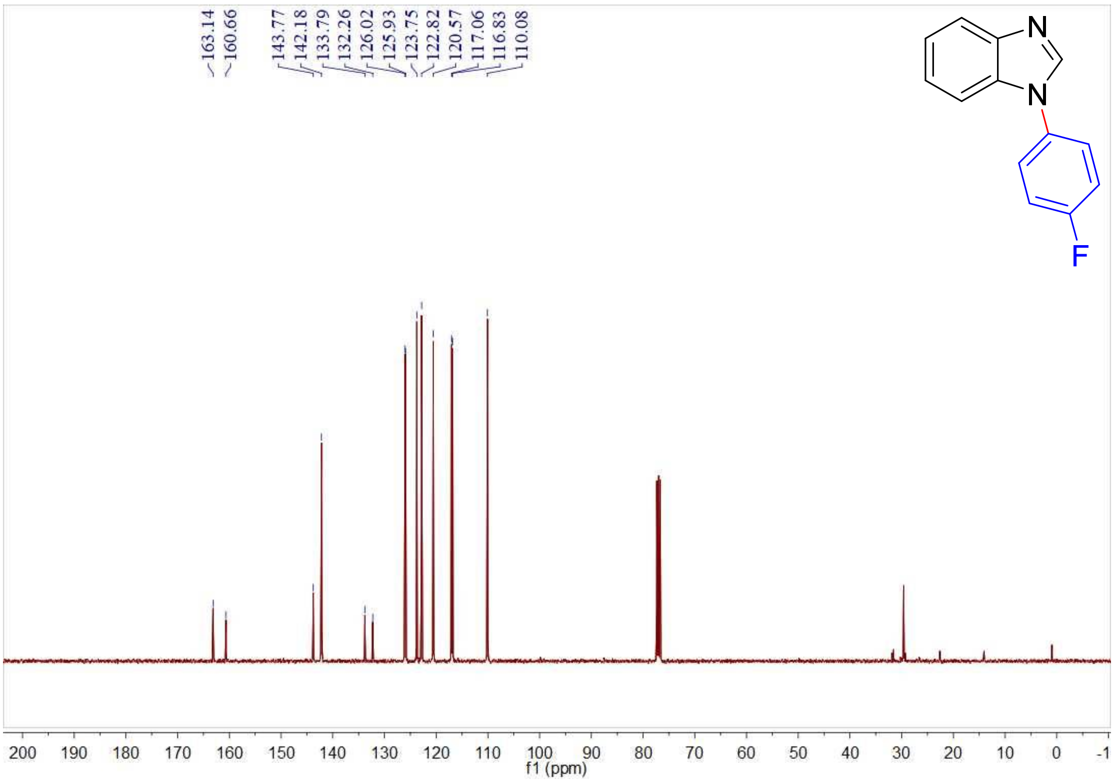

Figure S17. $^{13}C\{^{1}H\}$ NMR spectra of **8f**

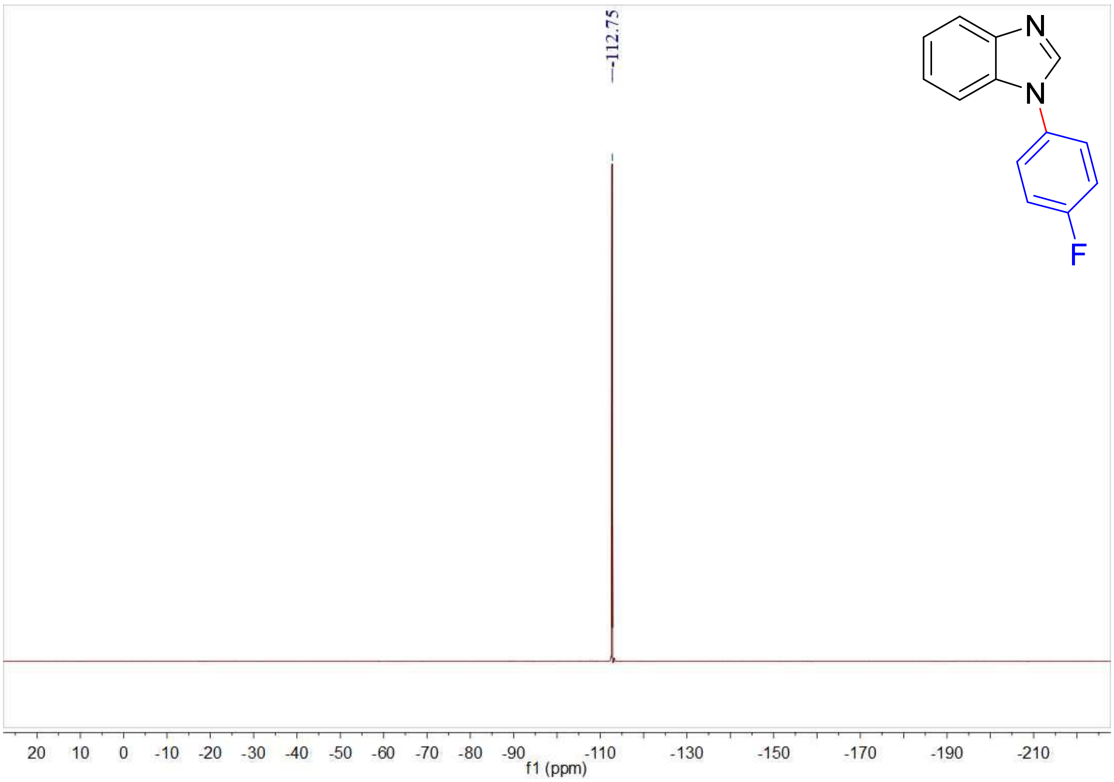

Figure S18. ^{19}F NMR spectra of **8f**

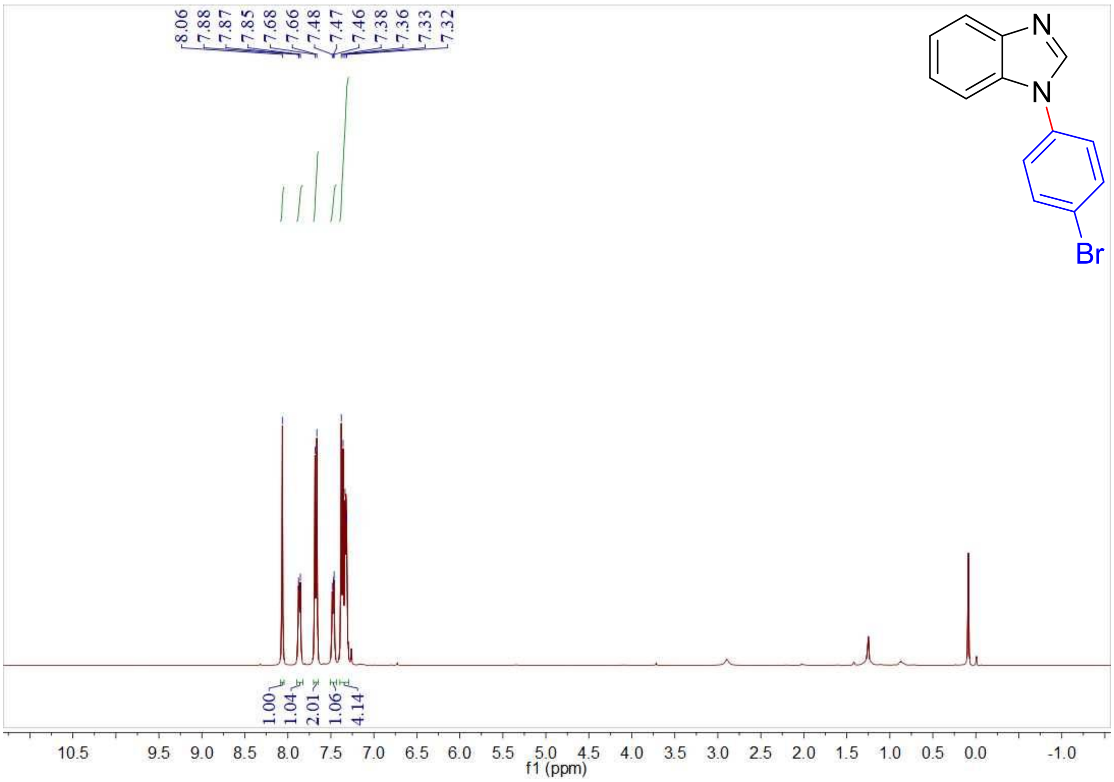

Figure S19. ^{1}H NMR spectra of **8g**

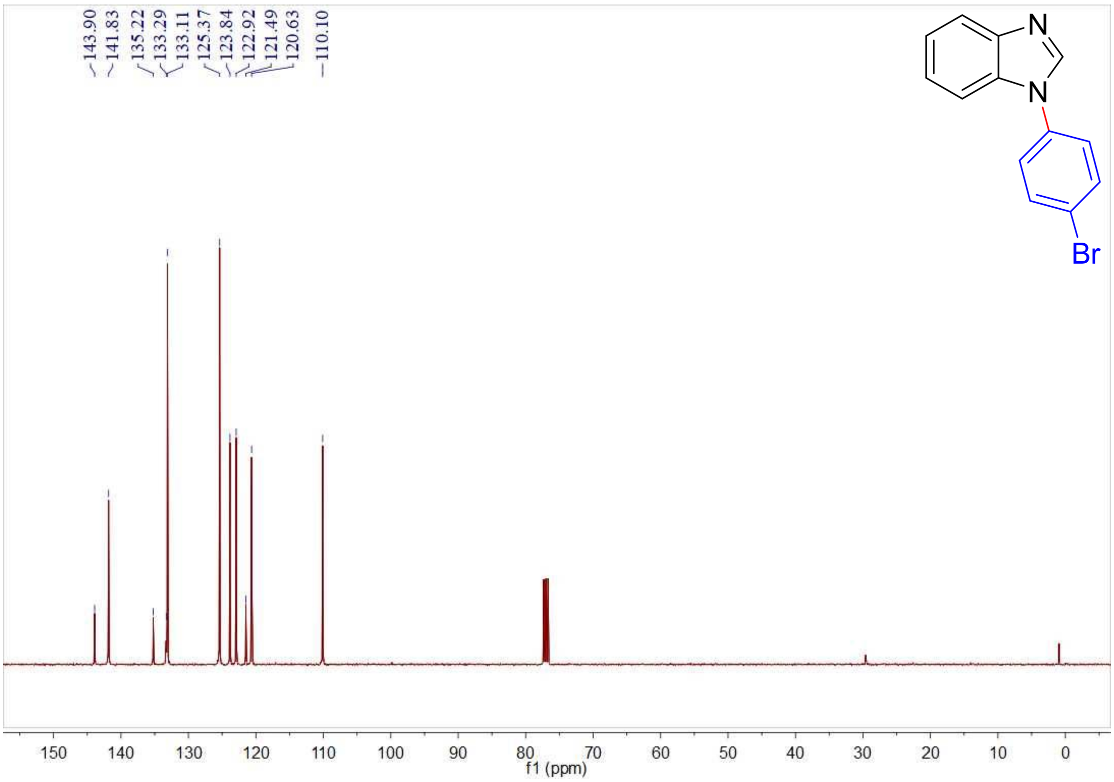

Figure S20. $^{13}C\{^{1}H\}$ NMR spectra of **8g**

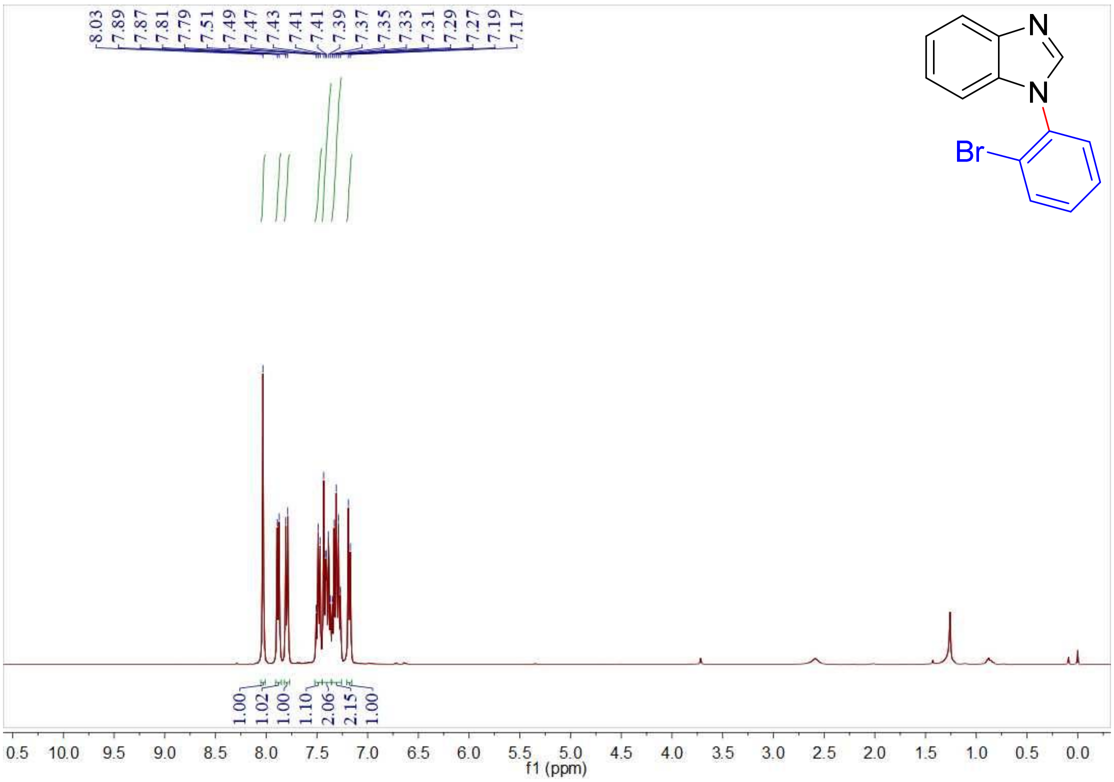

Figure S21. ^{1}H NMR spectra of **8h**

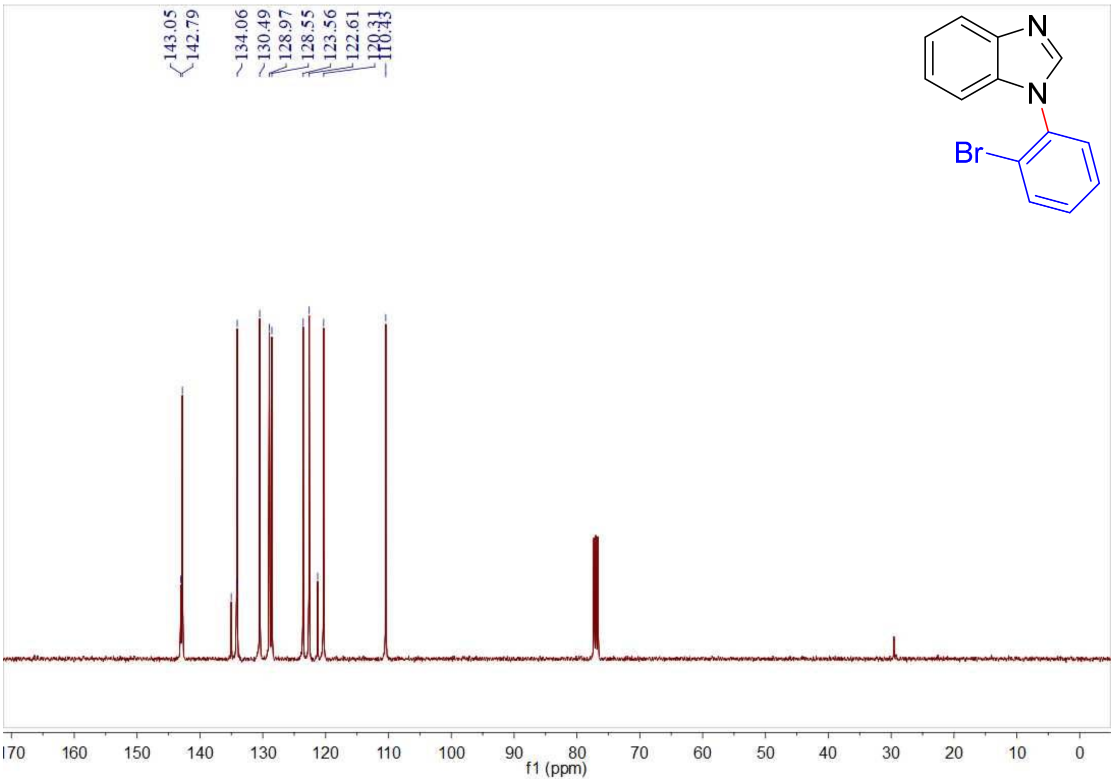

Figure S22. $^{13}C\{^{1}H\}$ NMR spectra of **8h**

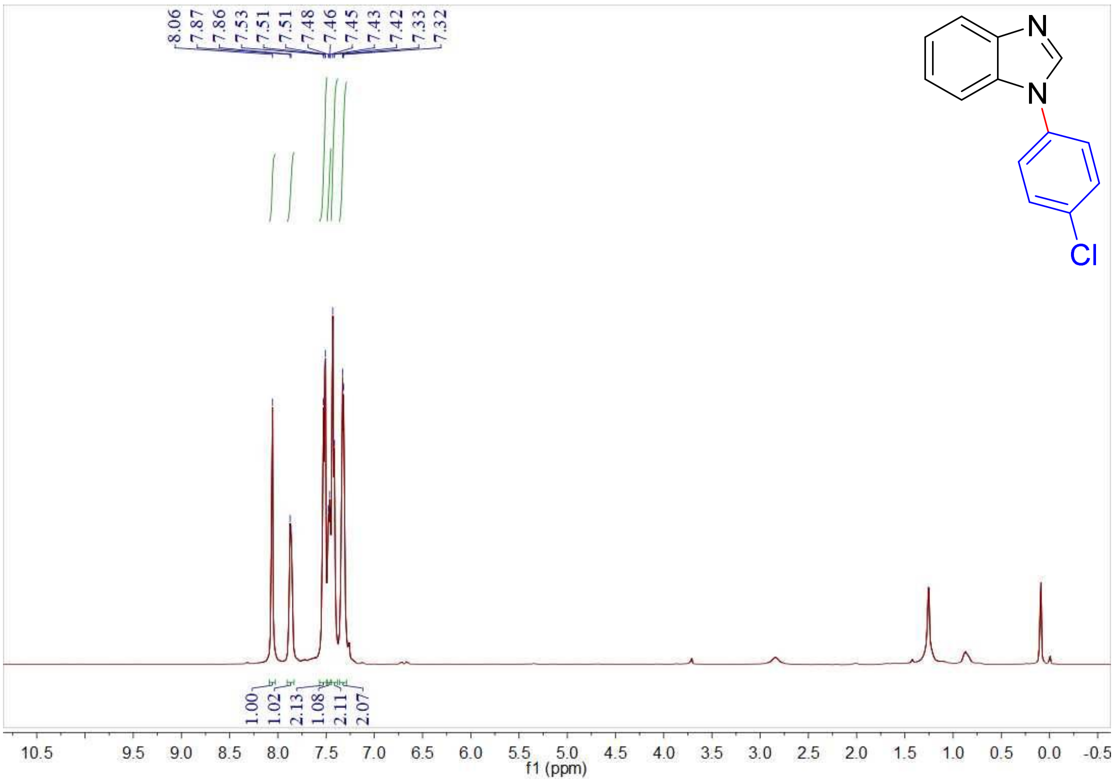

Figure S23. ^{1}H NMR spectra of **8i**

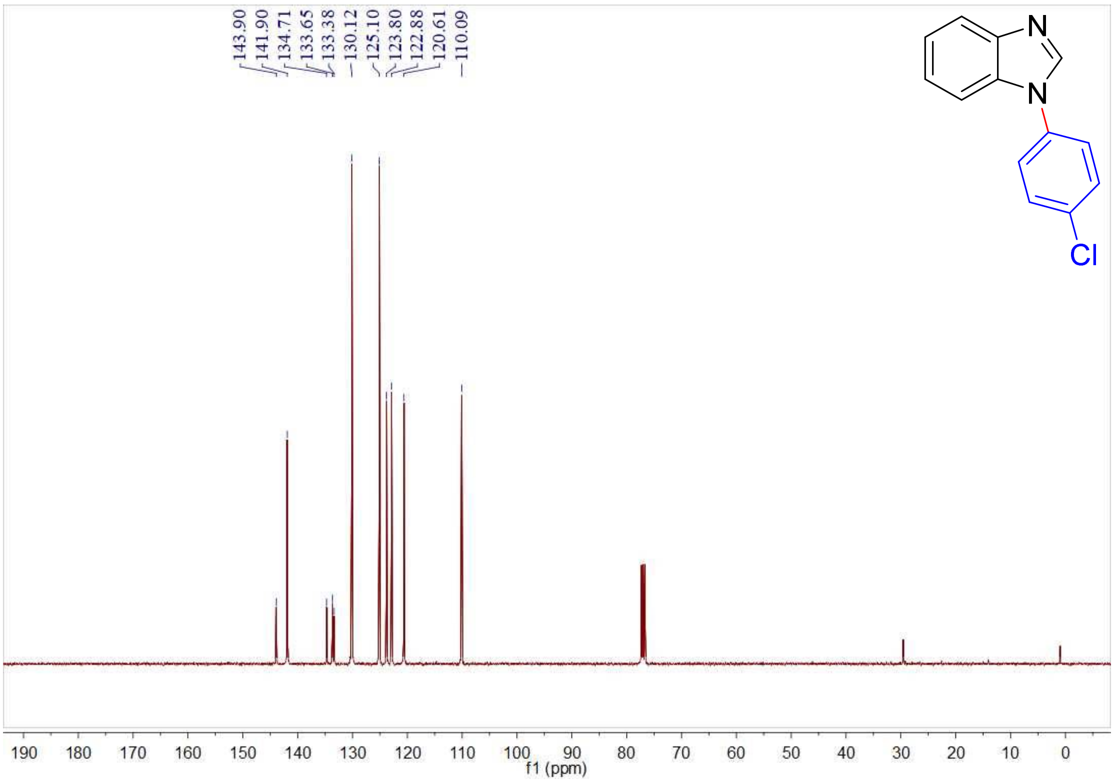

Figure S24. $^{13}C\{^{1}H\}$ NMR spectra of **8i**

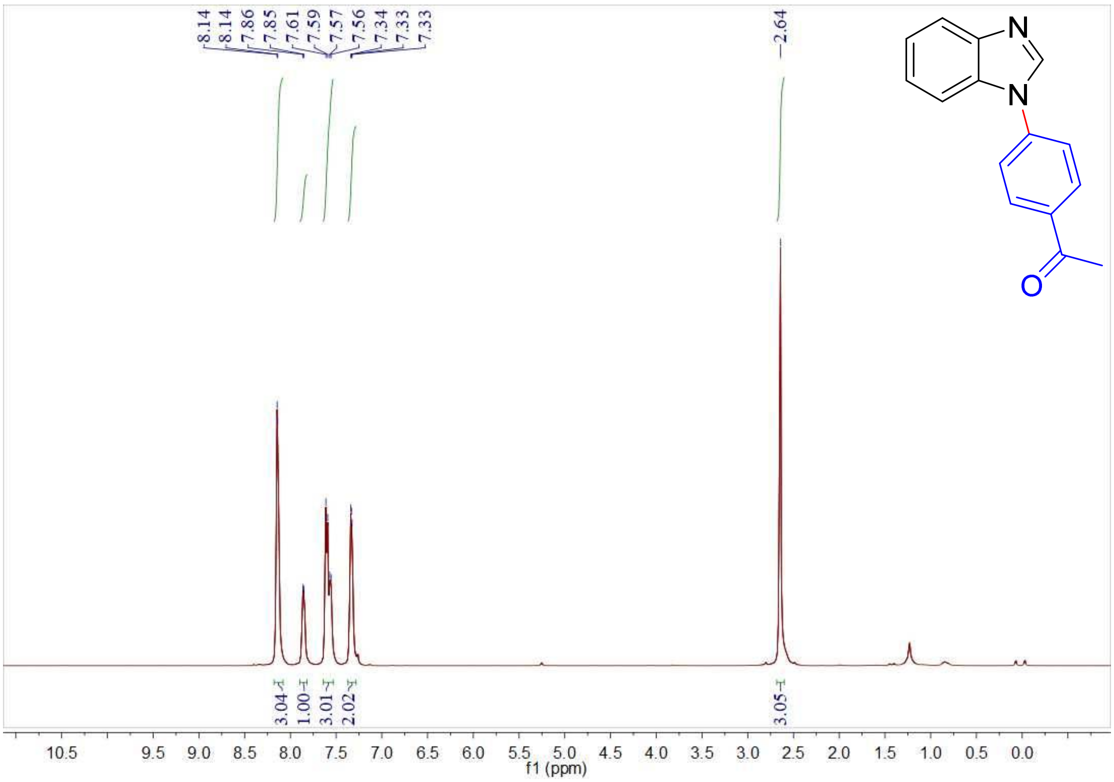

Figure S25. ^{1}H NMR spectra of **8j**

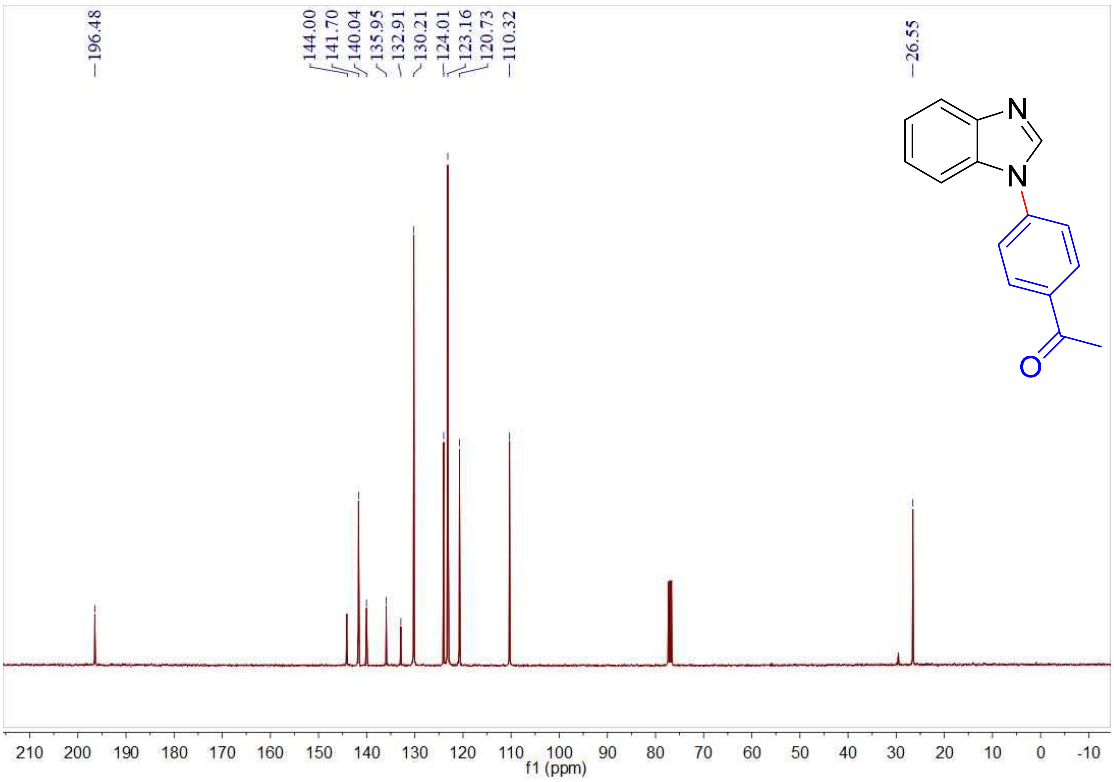

Figure S26. $^{13}C\{^{1}H\}$ NMR spectra of **8j**

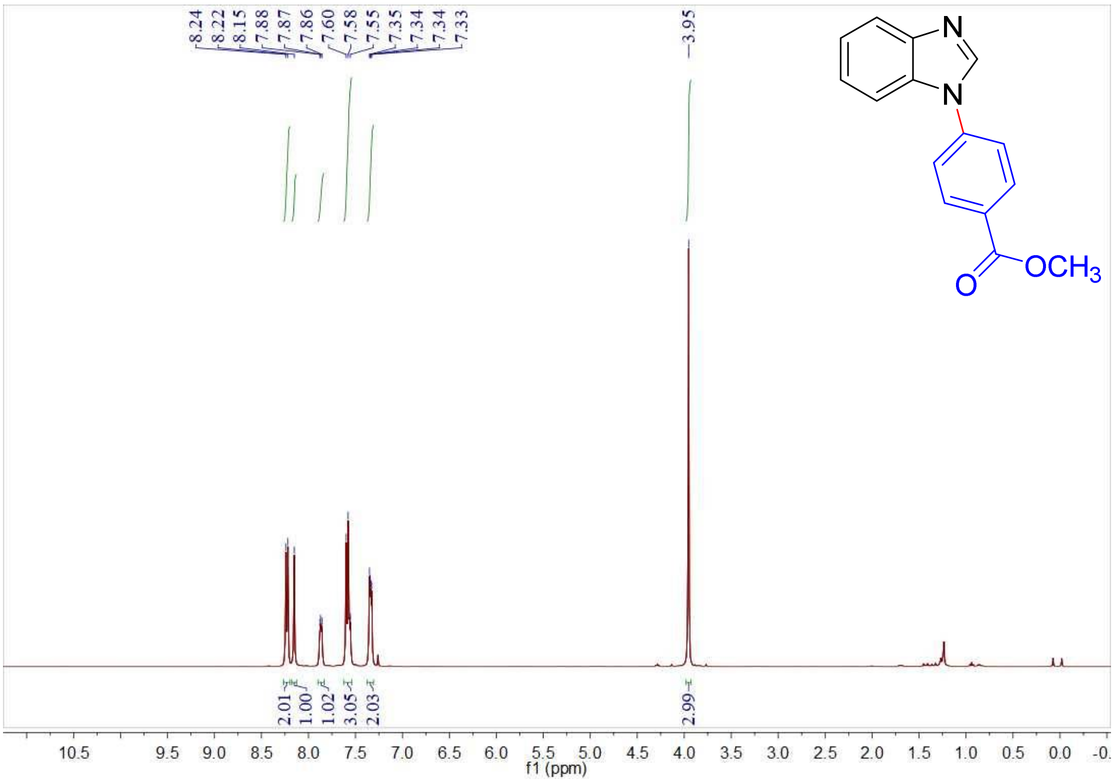

Figure S27. ^{1}H NMR spectra of **8k**

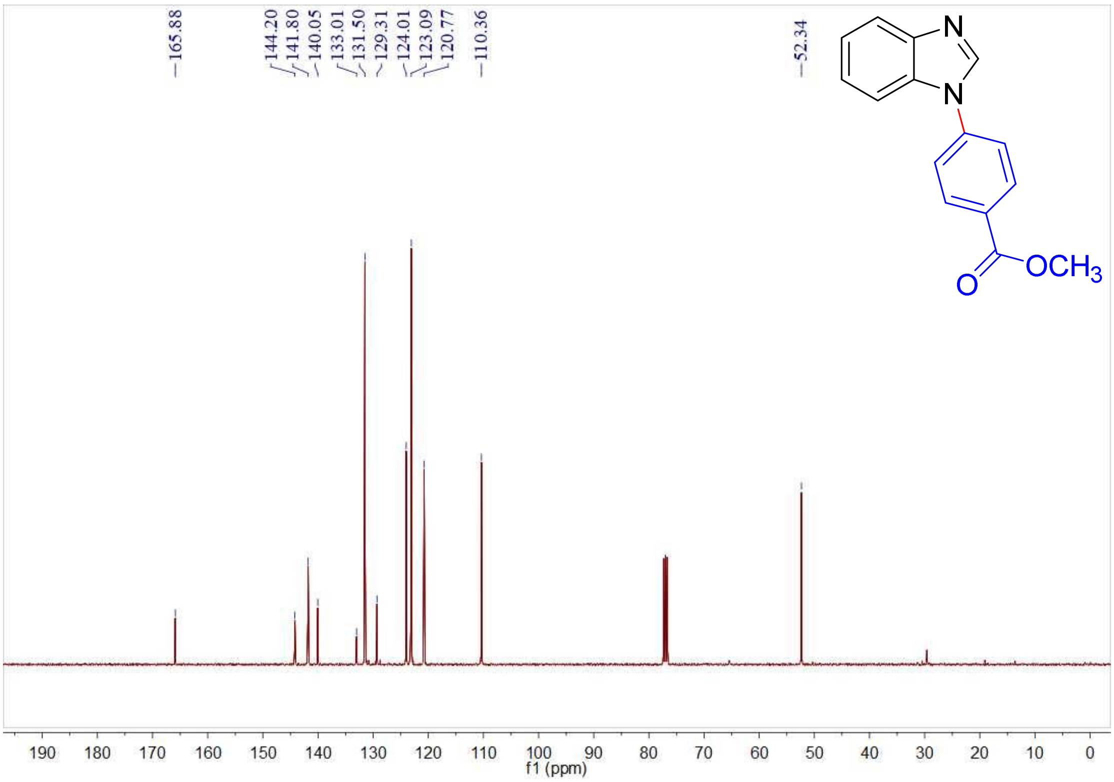

Figure S28. $^{13}C\{^{1}H\}$ NMR spectra of **8k**

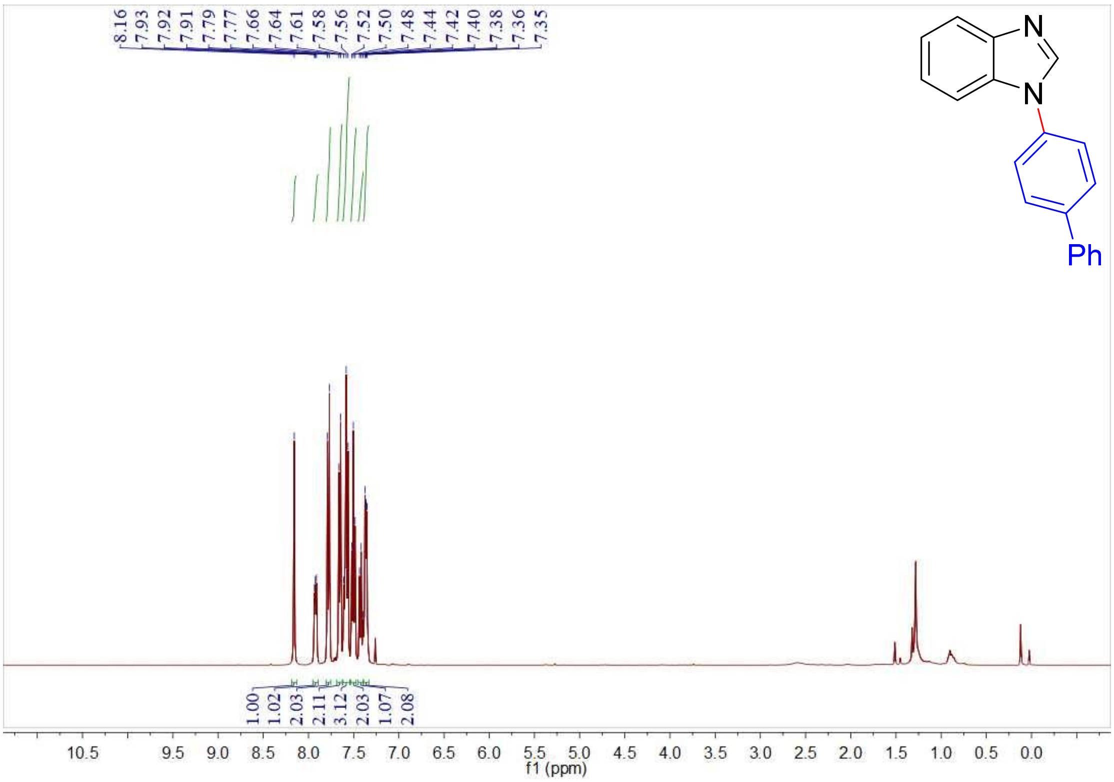

Figure S29. ^{1}H NMR spectra of **8l**

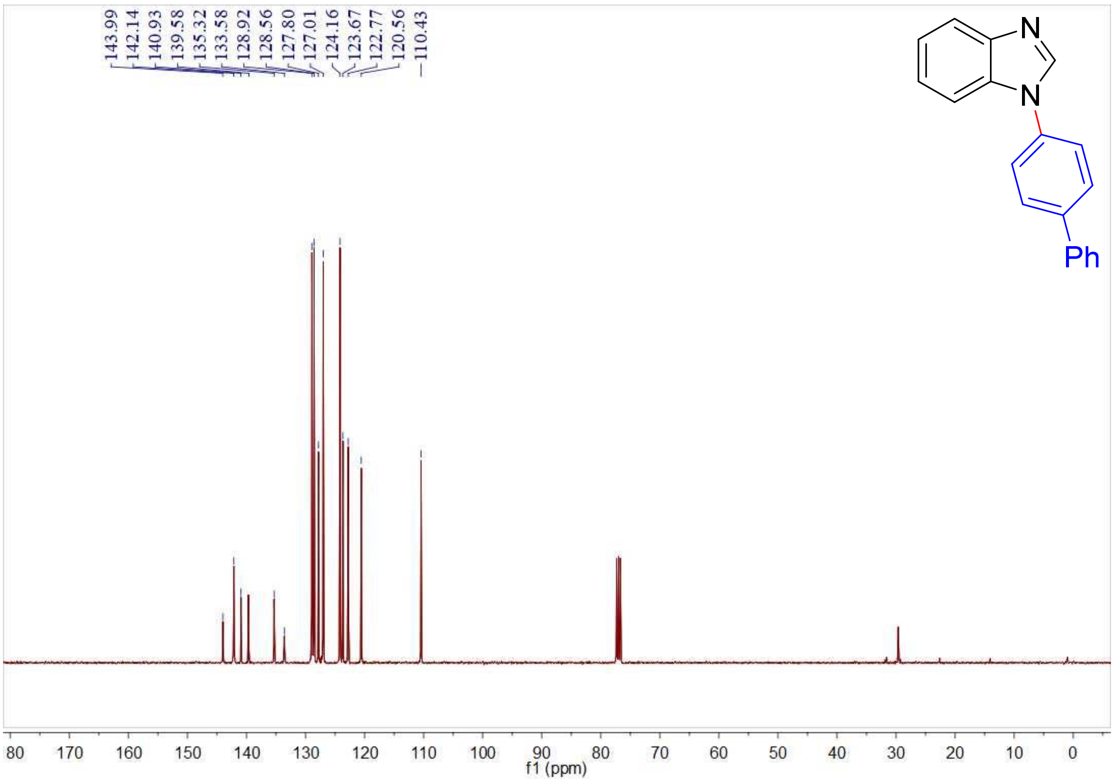

Figure S30. $^{13}C\{^{1}H\}$ NMR spectra of **8l**

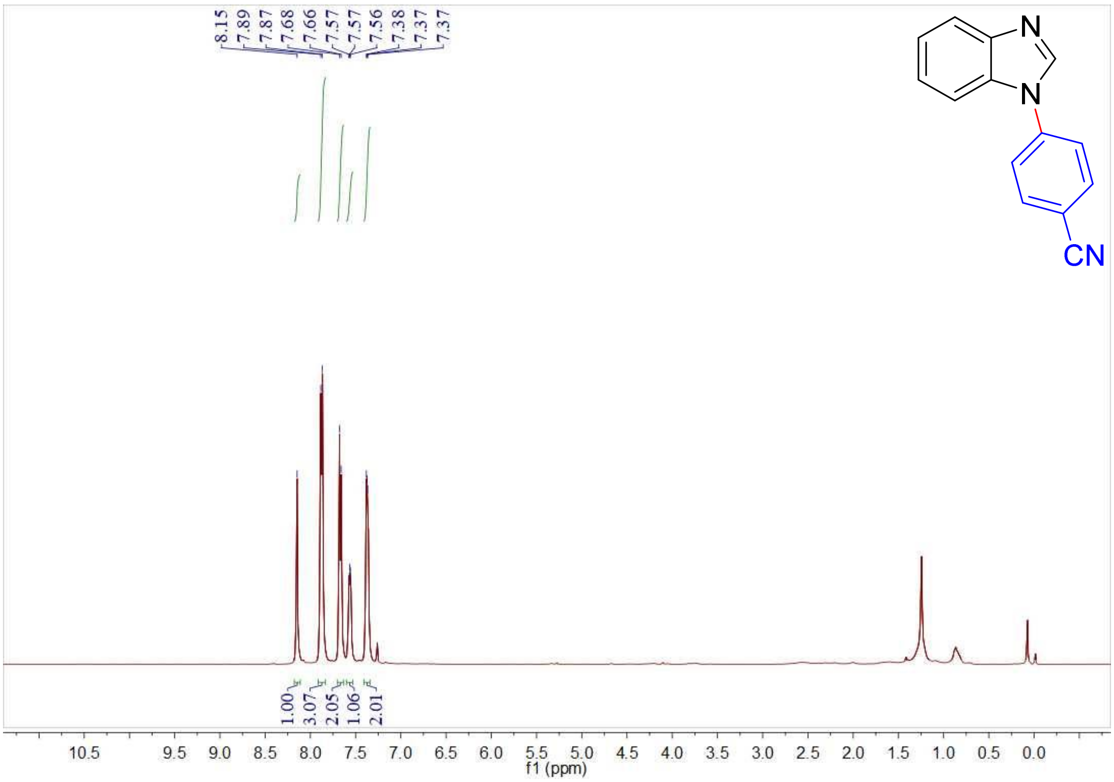

Figure S31. ^{1}H NMR spectra of **8m**

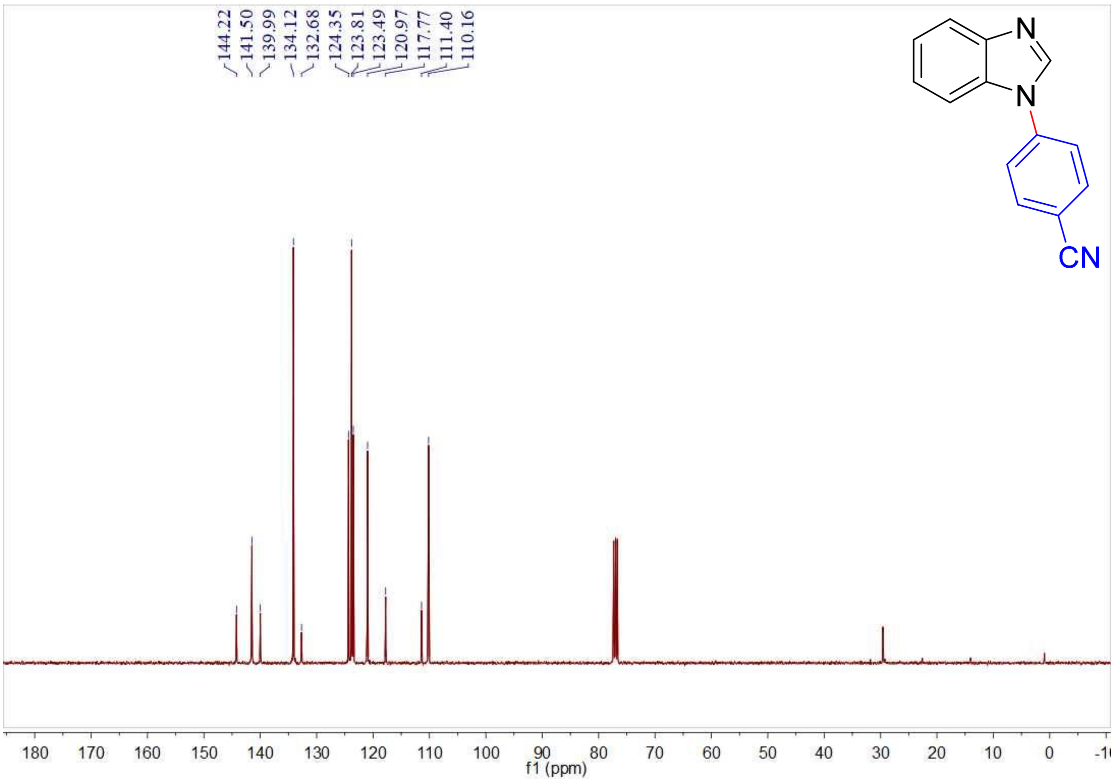

Figure S32. $^{13}C\{^{1}H\}$ NMR spectra of **8m**

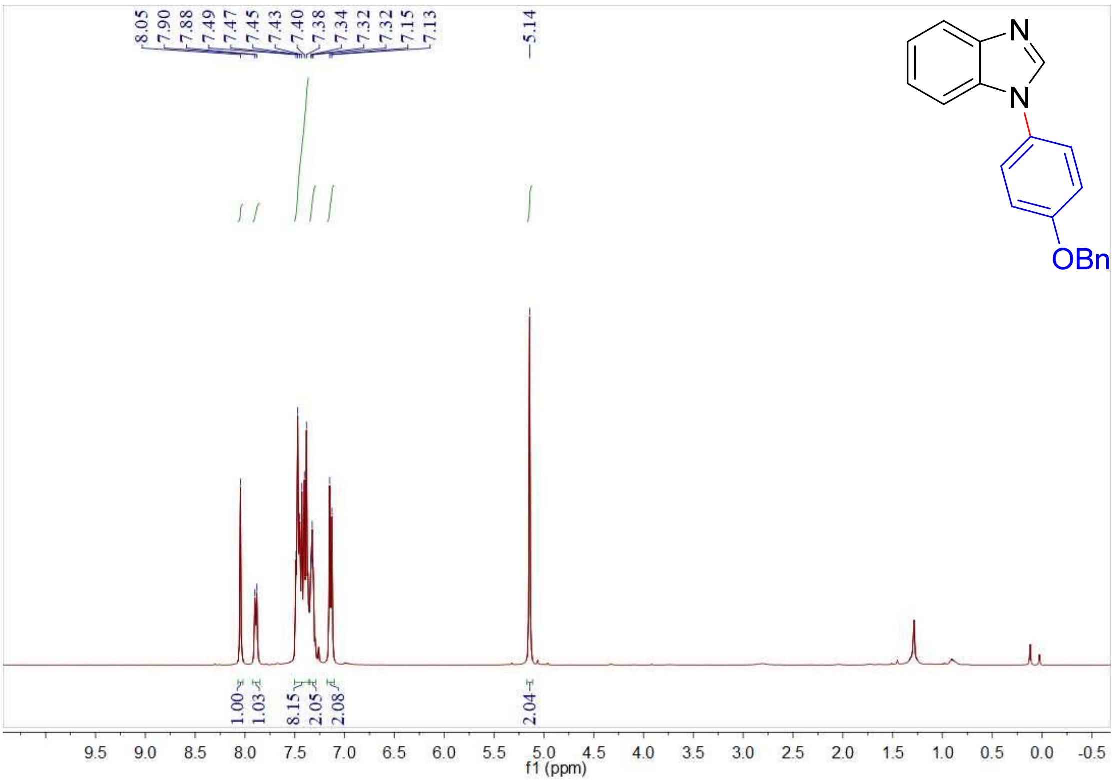

Figure S33. ^{1}H NMR spectra of **8n**

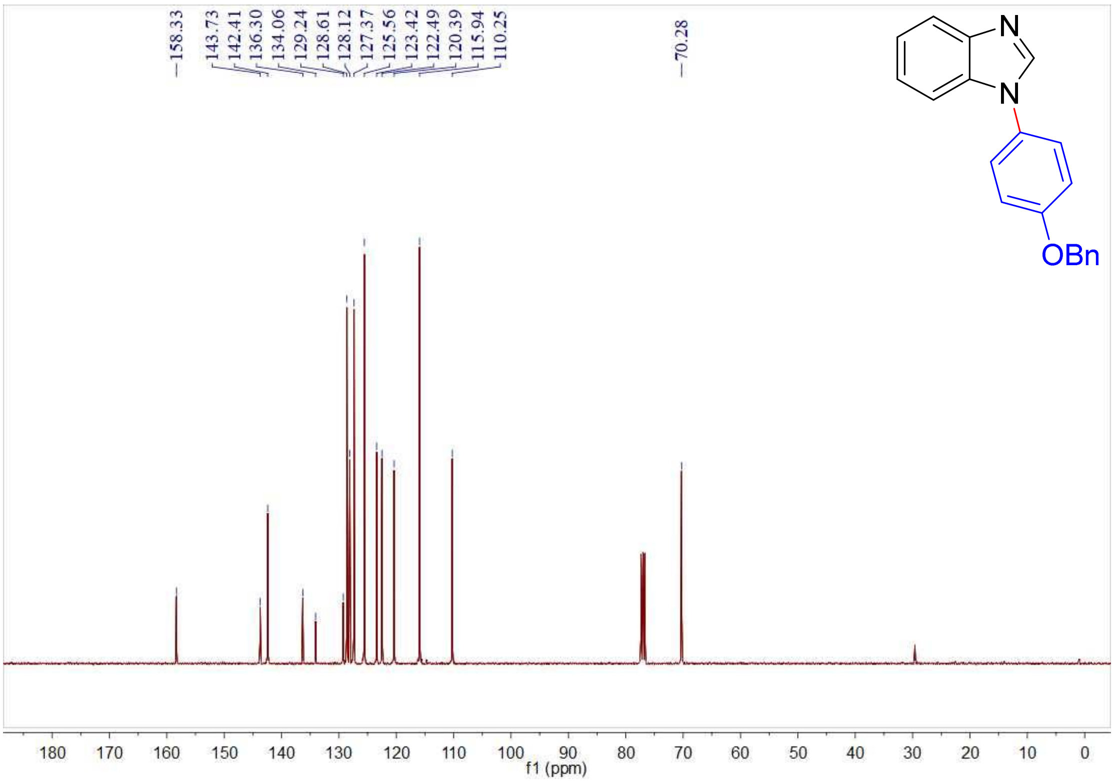

Figure S34. $^{13}C\{^{1}H\}$ NMR spectra of **8n**

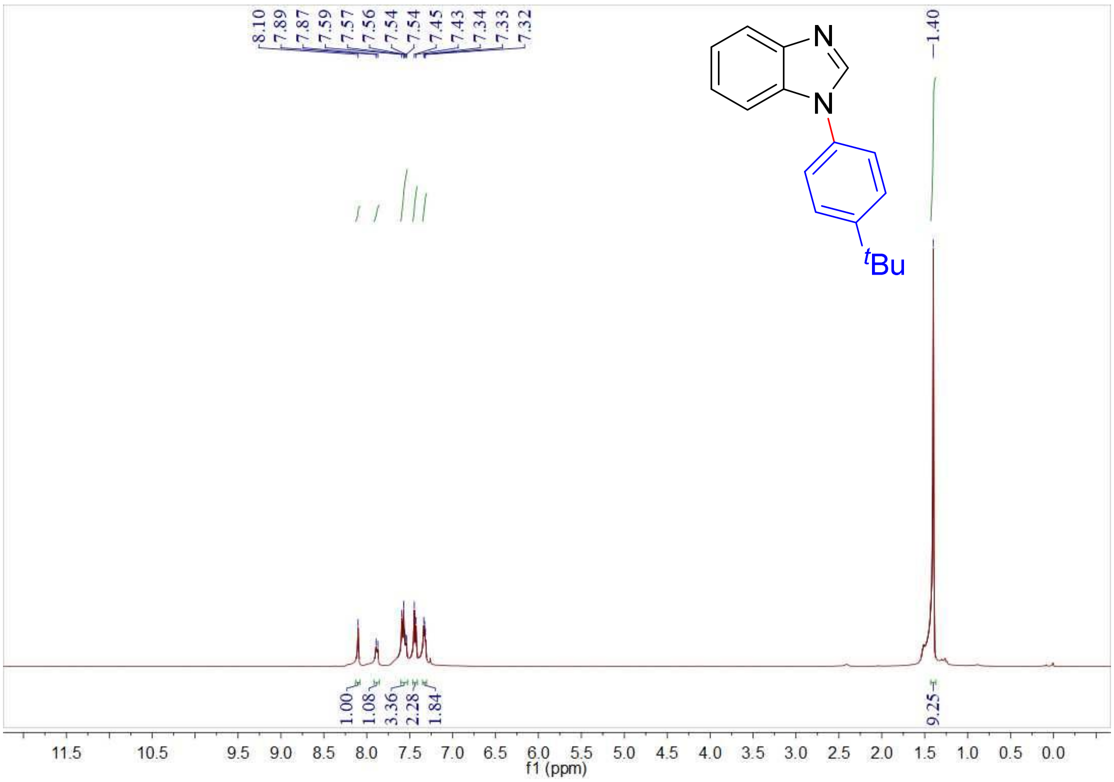

Figure S35. ^{1}H NMR spectra of **8o**

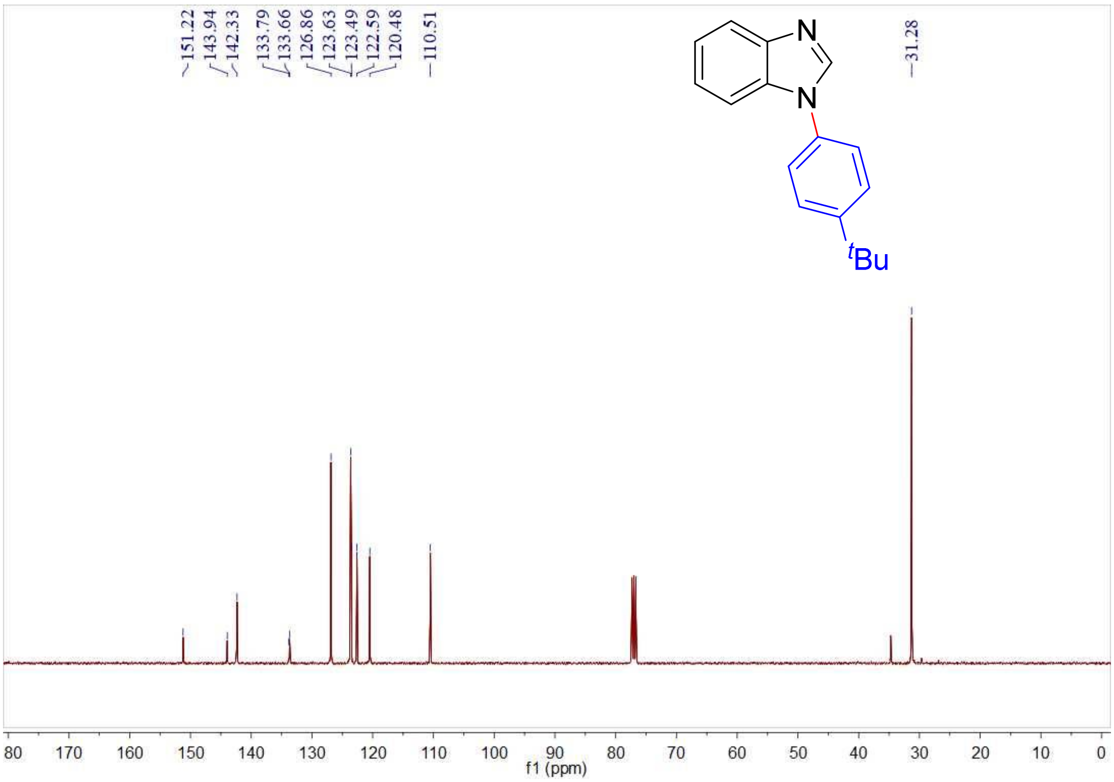

Figure S36. $^{13}C\{^{1}H\}$ NMR spectra of **8o**

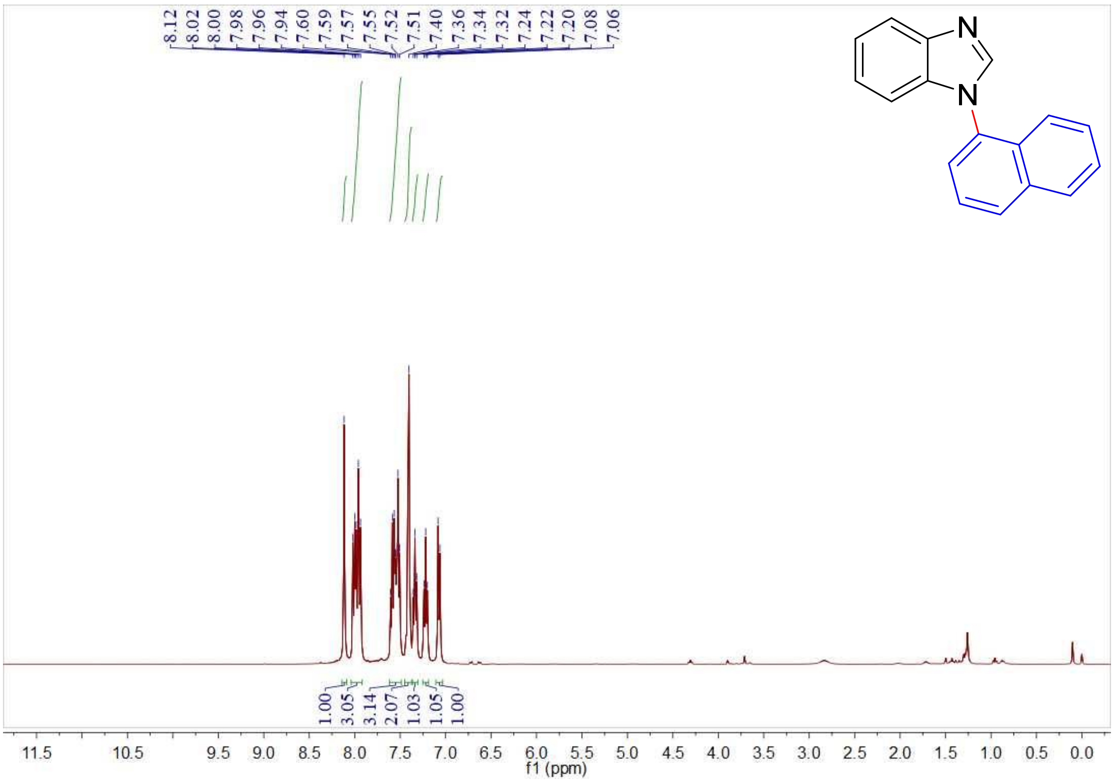

Figure S37. ^{1}H NMR spectra of **8p**

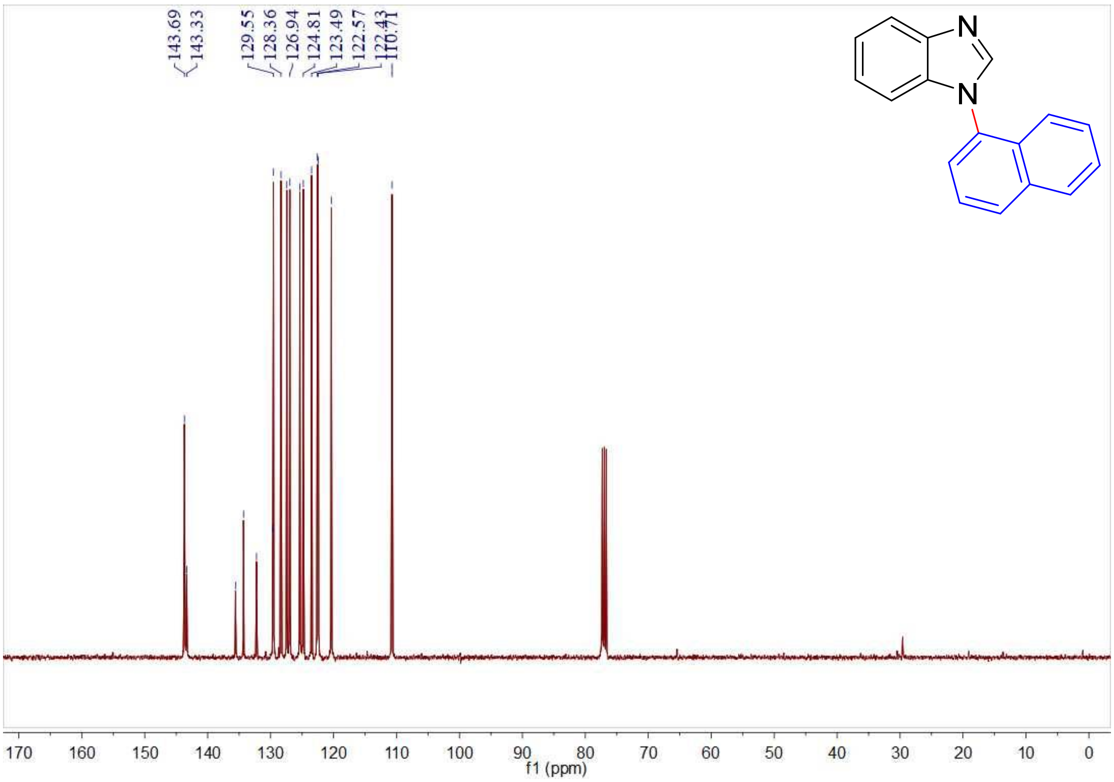

Figure S38. $^{13}C\{^{1}H\}$ NMR spectra of **8p**

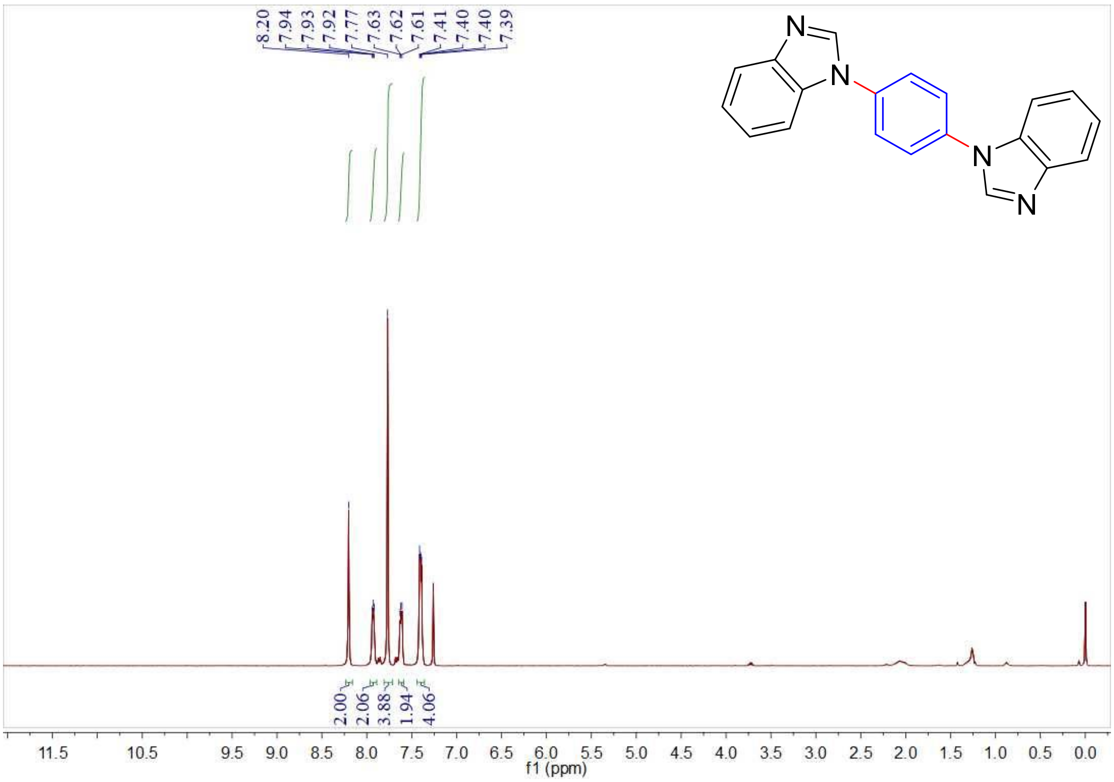

Figure S39. ^{1}H NMR spectra of **8s**

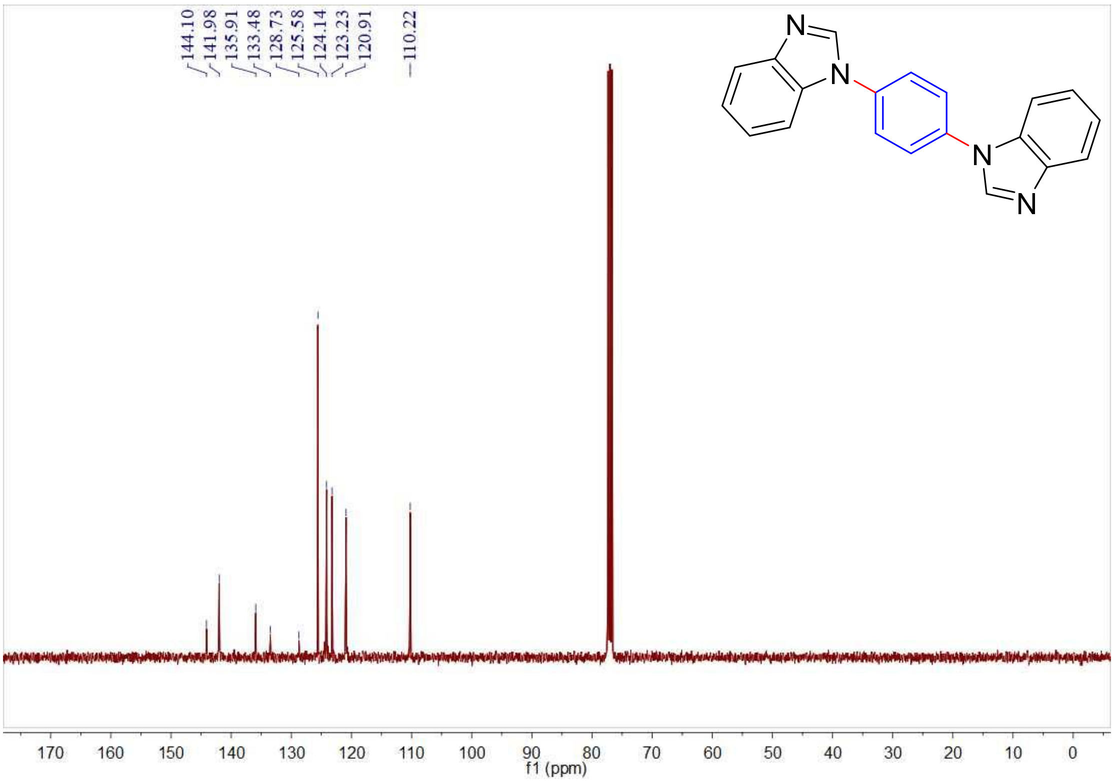

Figure S40. $^{13}C\{^{1}H\}$ NMR spectra of **8s**

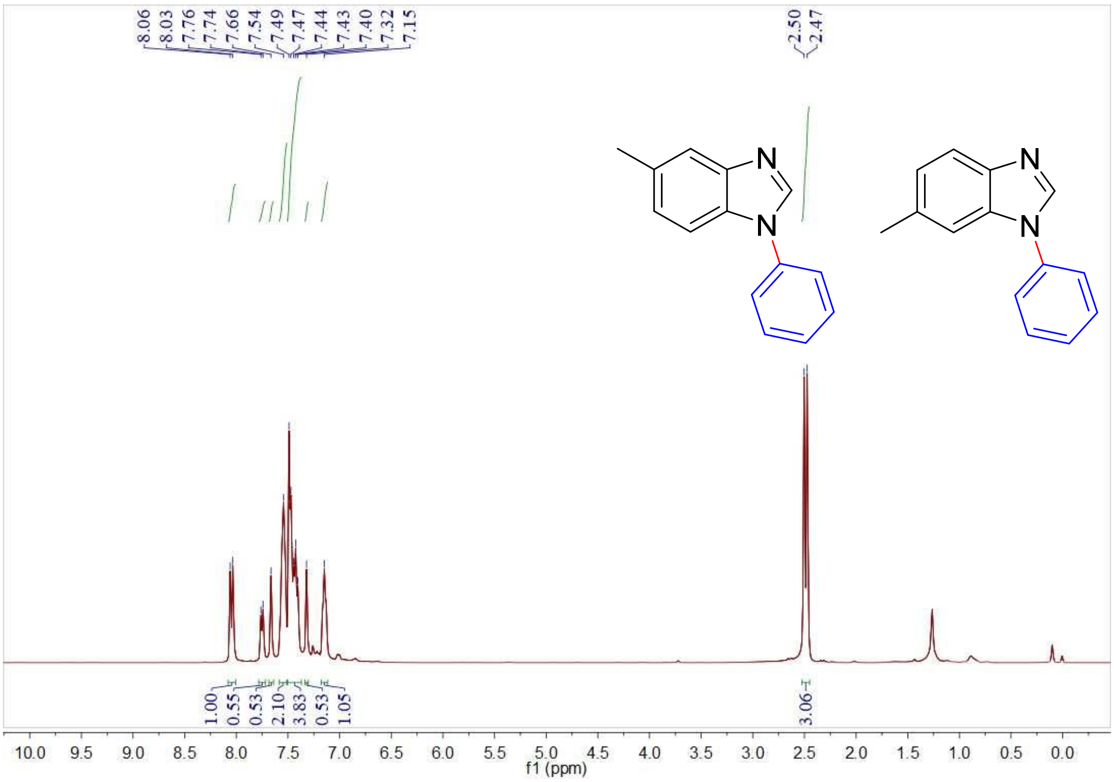

Figure S41. ^{1}H NMR spectra of **8t/8t'**

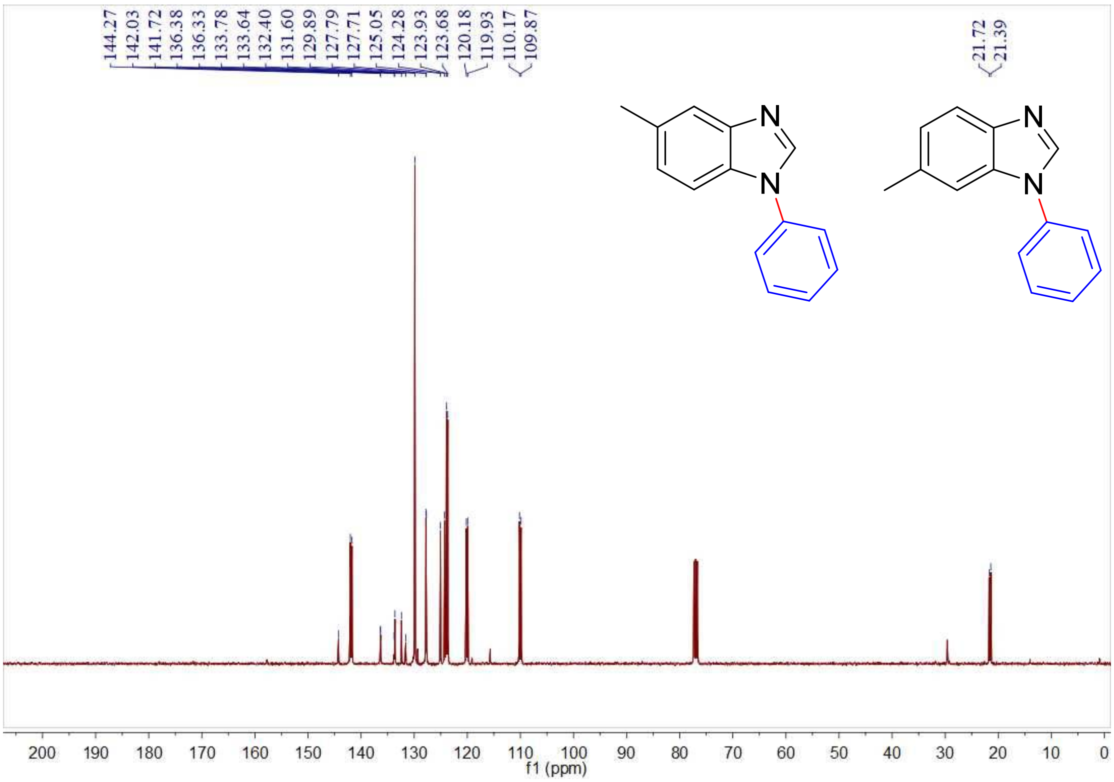

Figure S42. $^{13}C\{^{1}H\}$ NMR spectra of **8t/8t'**

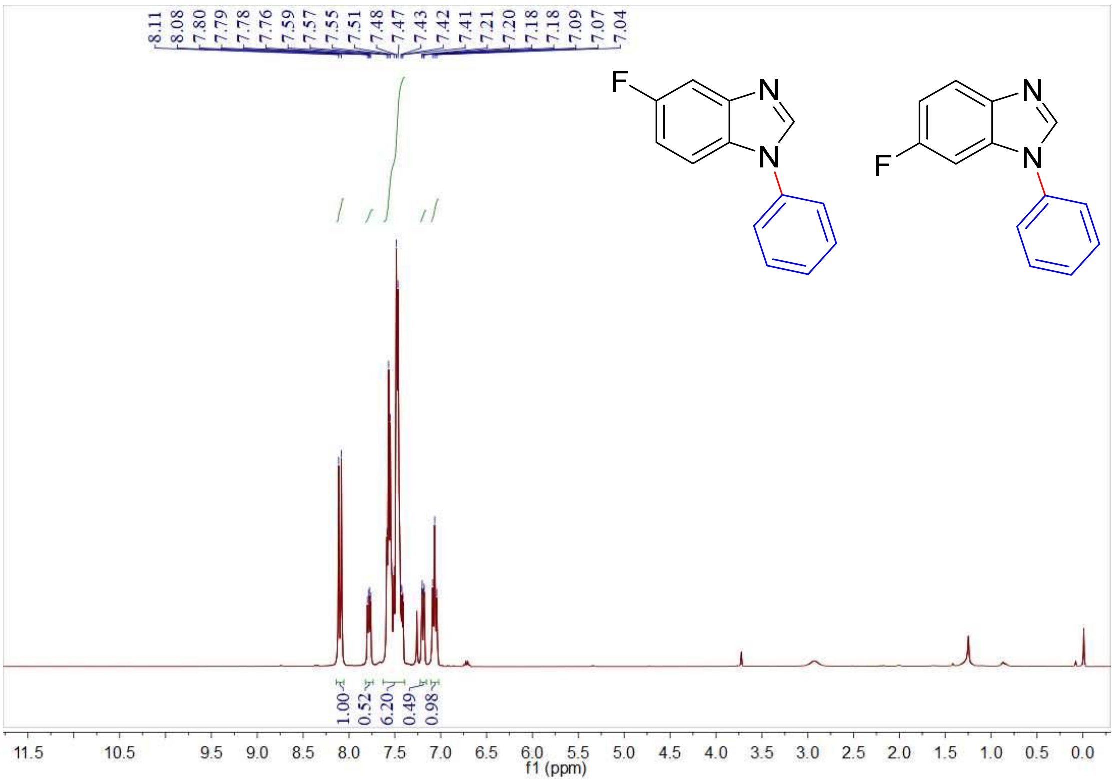

Figure S43. ^{1}H NMR spectra of **8u/8u'**

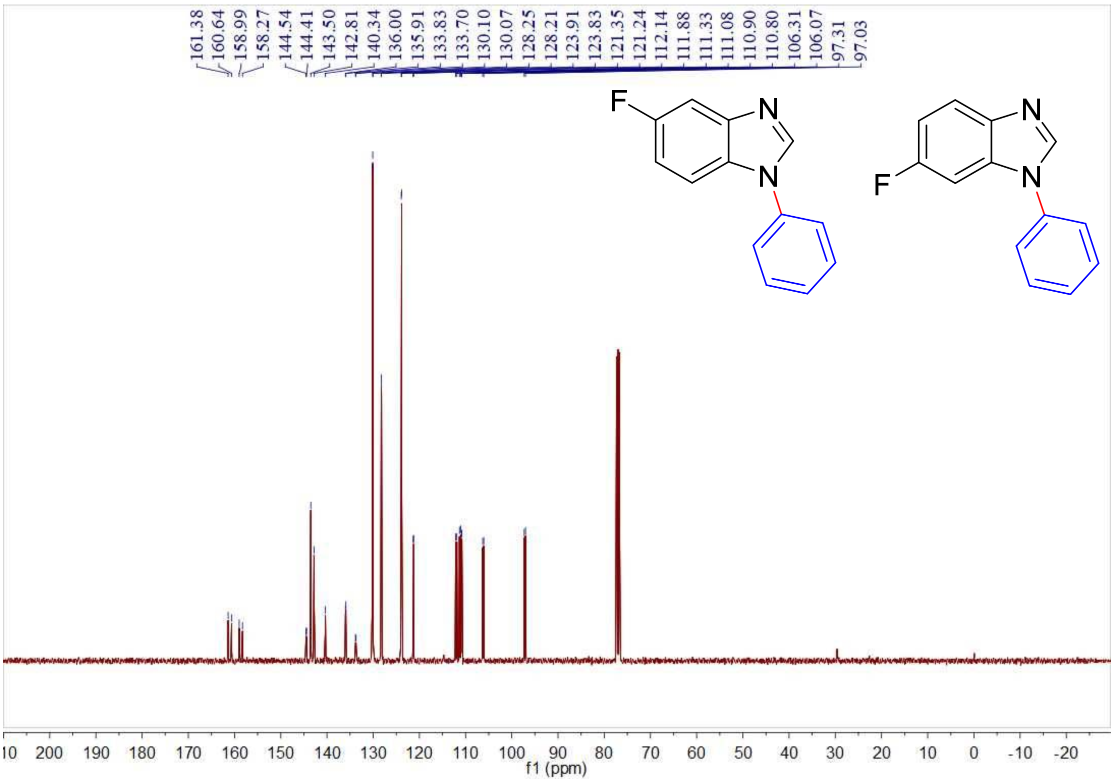

Figure S44. $^{13}C\{^{1}H\}$ NMR spectra of **8u/8u'**

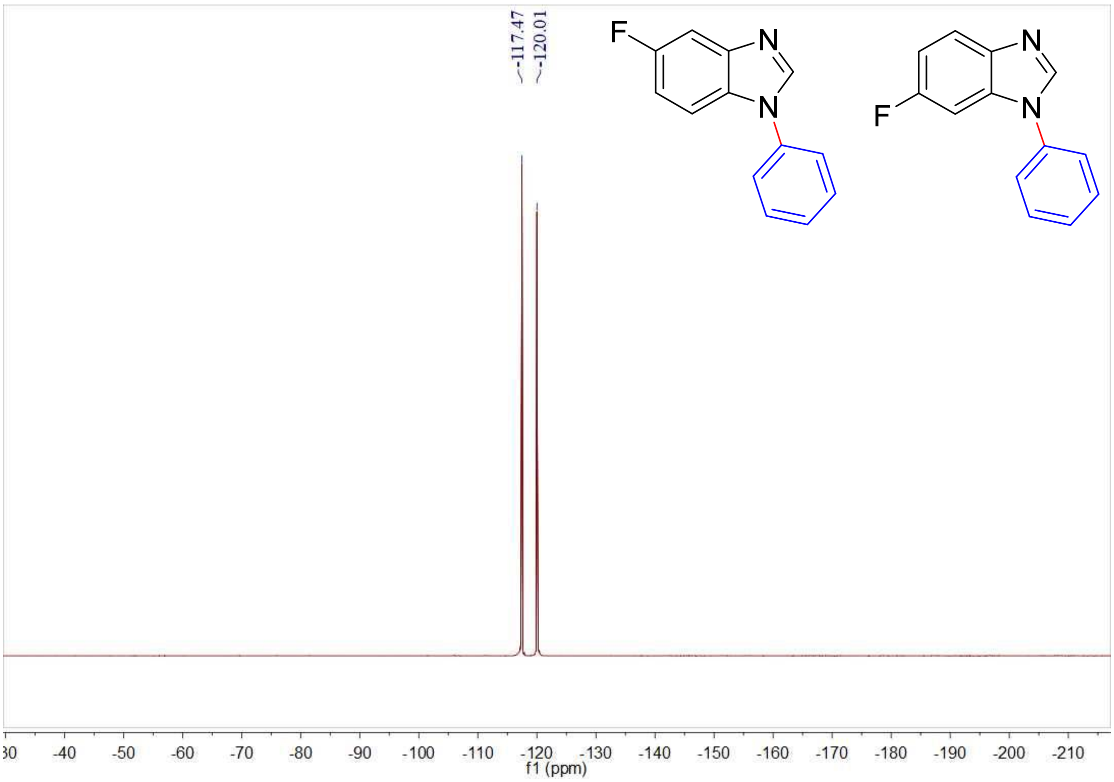

Figure S45. ^{19}F NMR spectra of **8u/8u'**

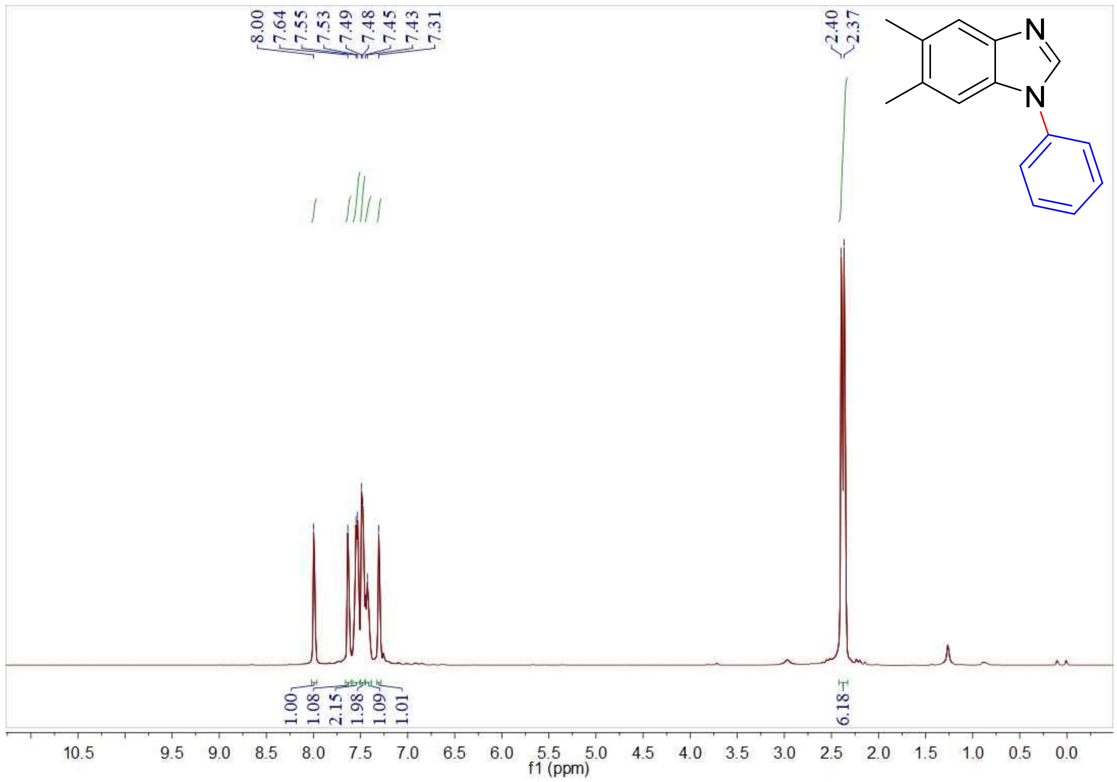

Figure S46. ^{1}H NMR spectra of **8v**

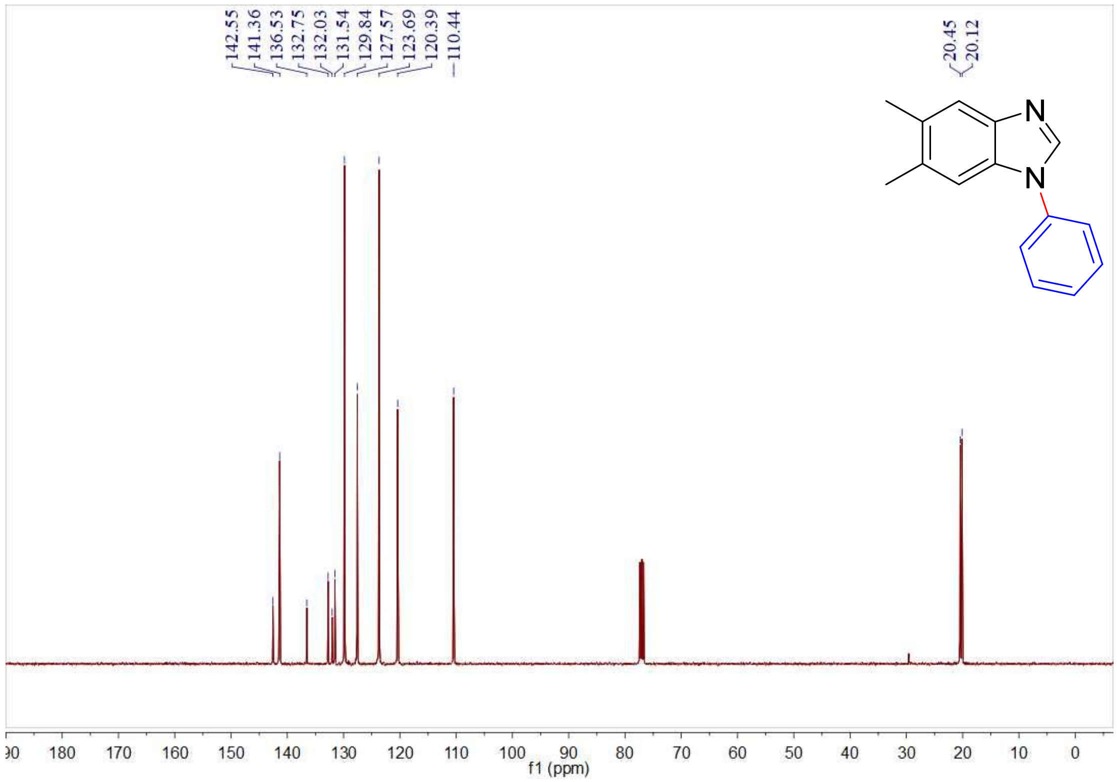

Figure S47. $^{13}C\{^{1}H\}$ NMR spectra of **8v**

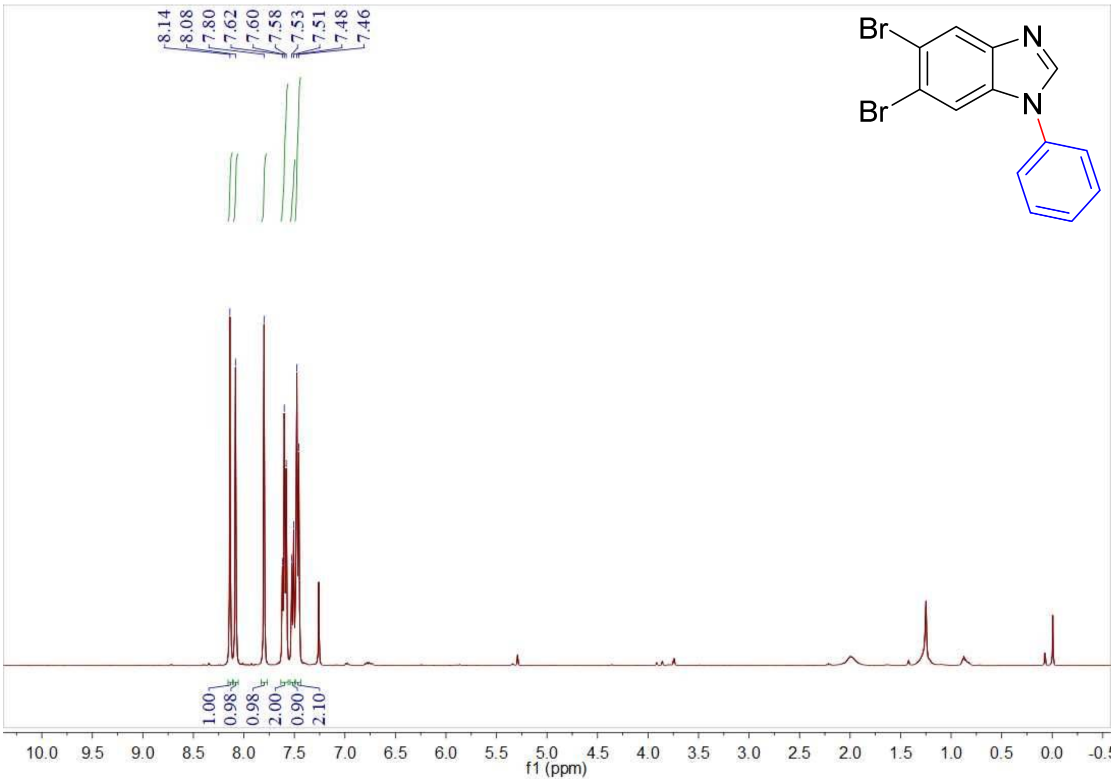

Figure S48. ^{1}H NMR spectra of **8w**

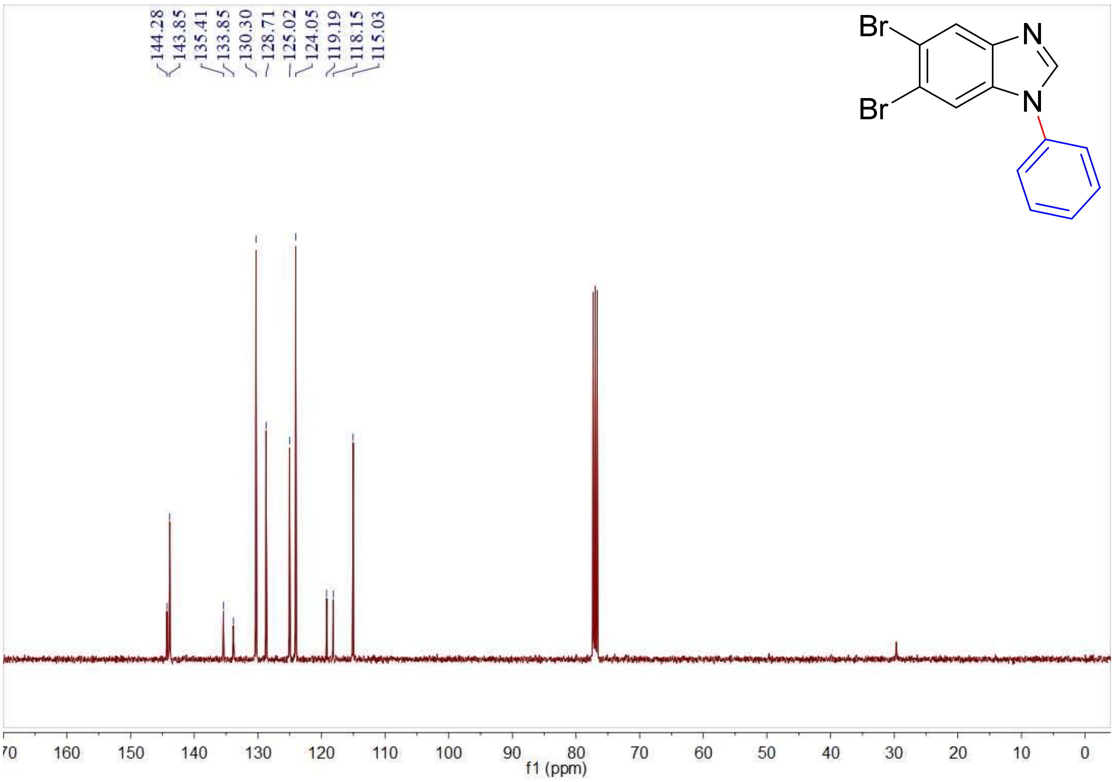

Figure S49. $^{13}C\{^{1}H\}$ NMR spectra of **8w**

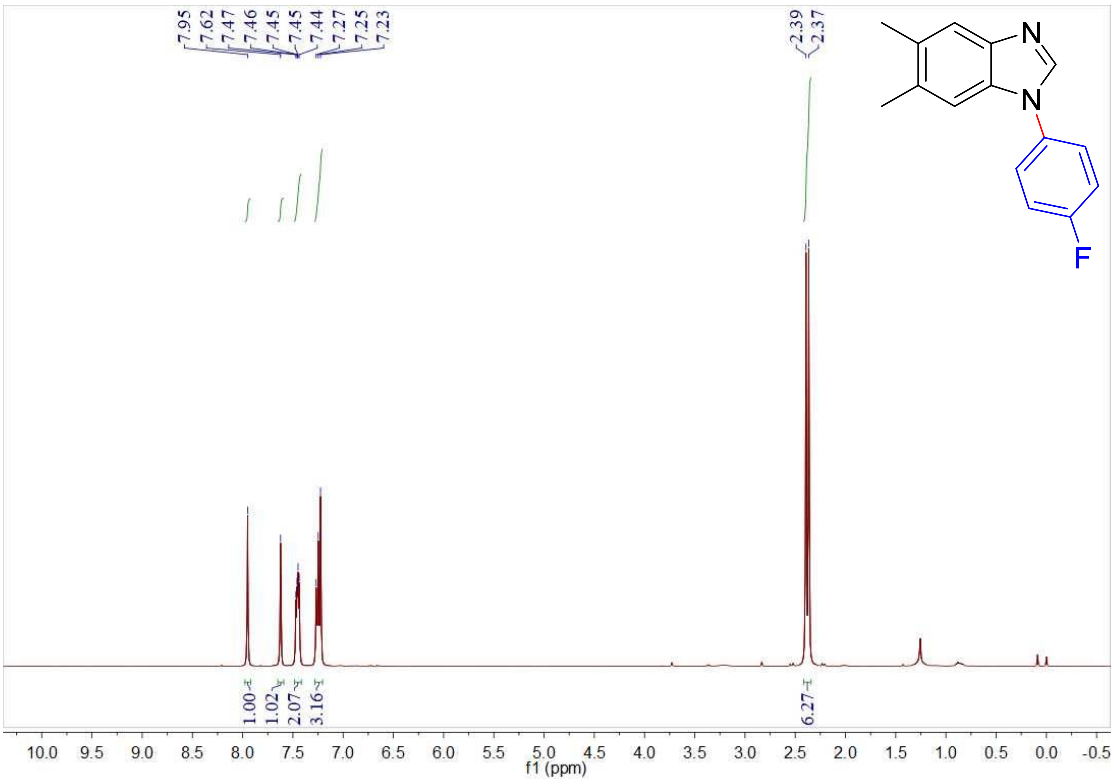

Figure S50. ^{1}H NMR spectra of **8x**

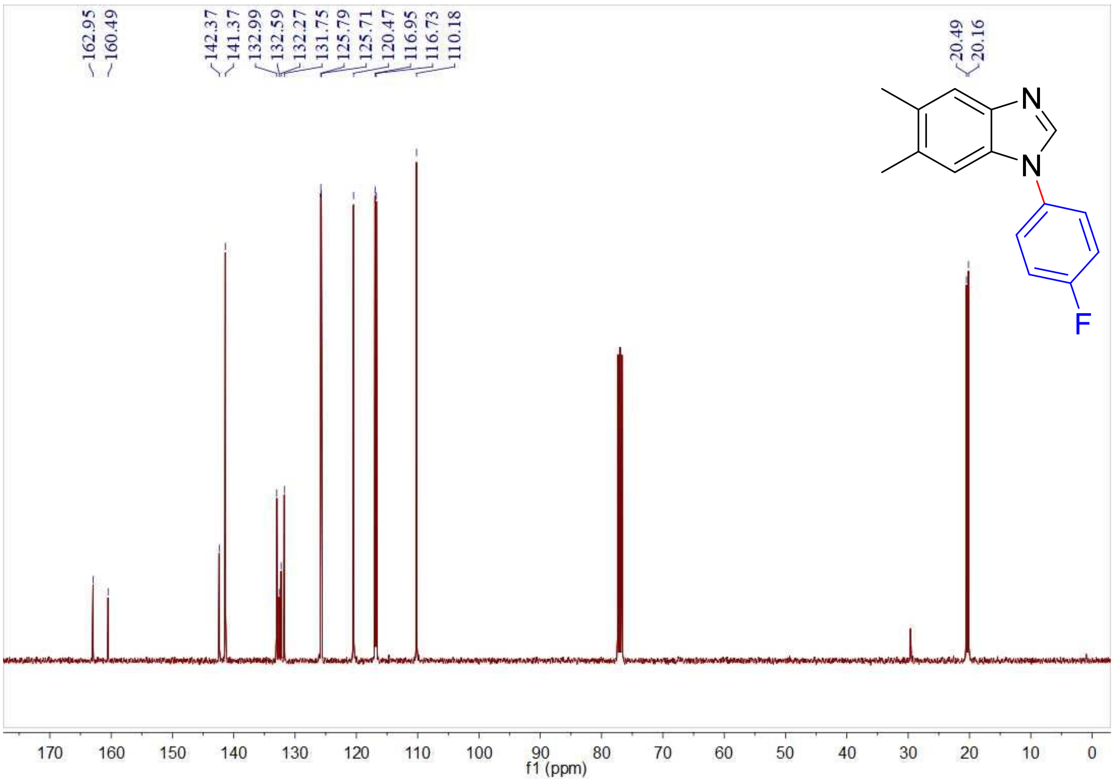

Figure S51. $^{13}C\{^{1}H\}$ NMR spectra of **8x**

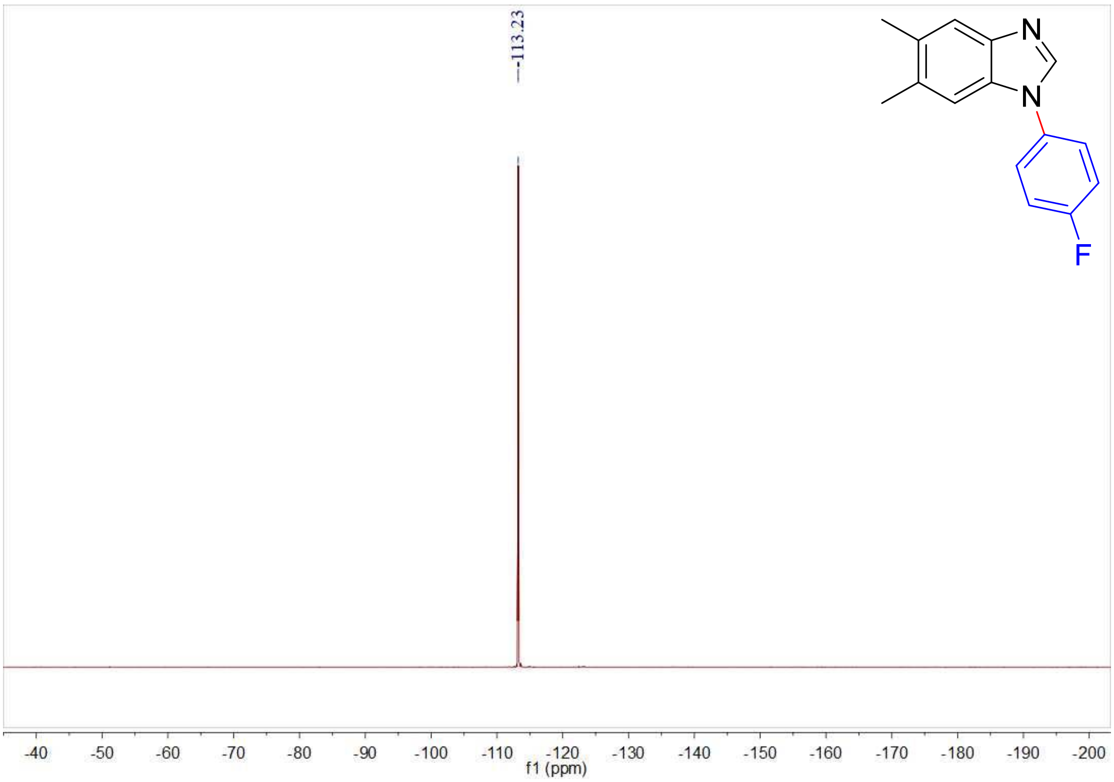

Figure S52. ^{19}F NMR spectra of **8x**

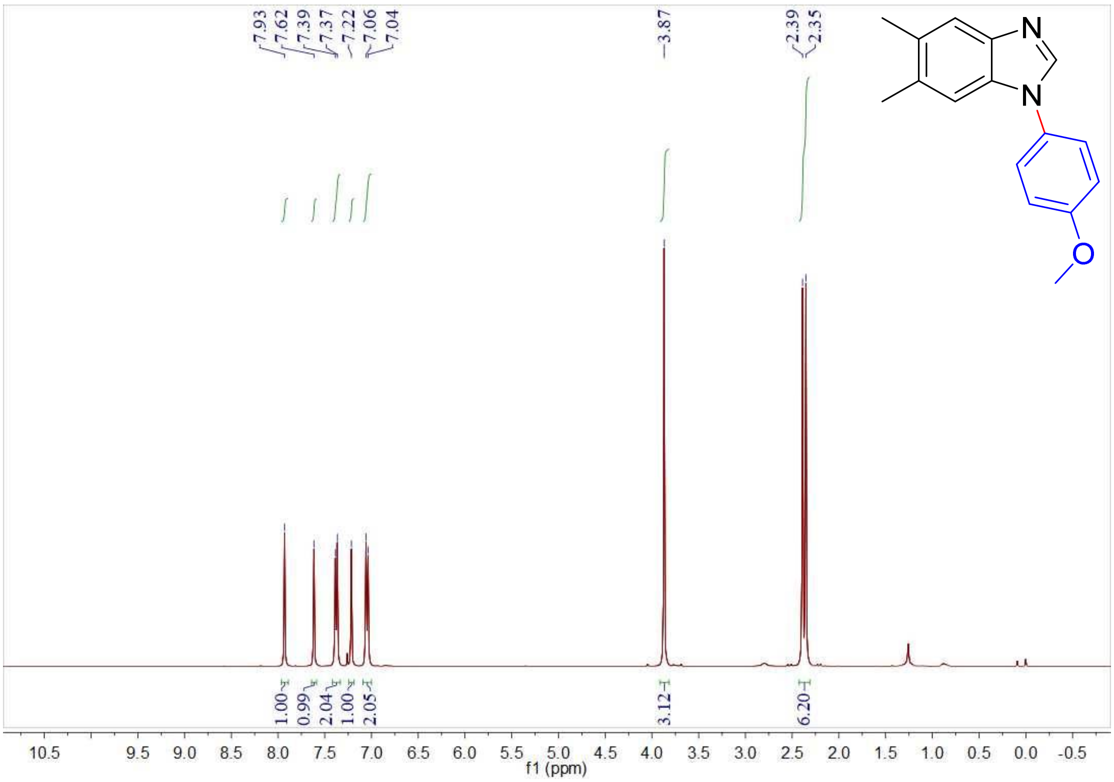

Figure S53. ^{1}H NMR spectra of **8y**

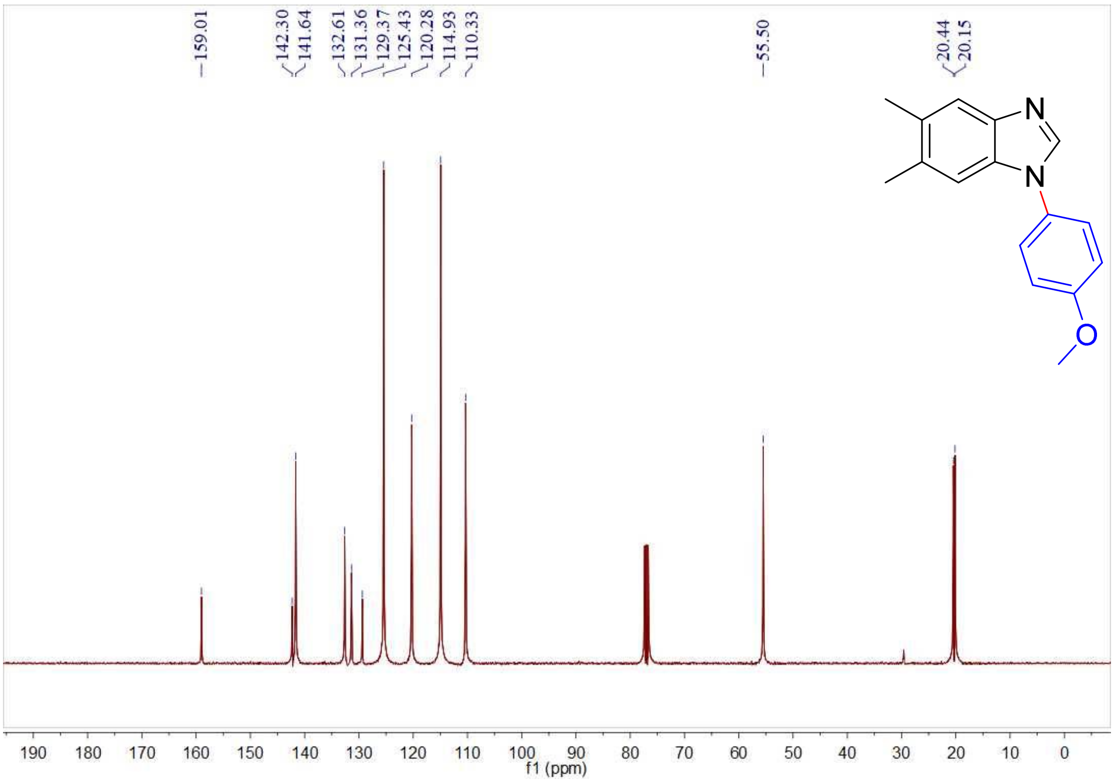

Figure S54. $^{13}C\{^{1}H\}$ NMR spectra of **8y**
